# Geochemical evolution of dissolved trace elements in space and time in the Ramganga River, India

**DOI:** 10.1007/s10661-023-11665-0

**Published:** 2023-09-05

**Authors:** Indra Sekhar Sen, Sarwar Nizam, Aqib Ansari, Michael Bowes, Bharat Choudhary, Miriam Glendell, Surajit Ray, Marian Scott, Claire Miller, Craig Wilkie, Rajiv Sinha

**Affiliations:** 1 Department of Earth Sciences, Indian Institute of Technology Kanpur, Kanpur, India; 2 Physical Research Laboratory, Navrangpura, Ahmedabad 380009 India; 3 UK Centre for Ecology and Hydrology, OX10 8BB Wallingford, UK; 4 The James Hutton Institute, Aberdeen, AB15 8QH UK; 5 School of Mathematics and Statistics, University of Glasgow, Glasgow, G128QQ UK

**Keywords:** River geochemistry, Ramganga River, Ganga rivers, Metal pollution, Human impact

## Abstract

Understanding the spatiotemporal dynamics of river water chemistry from its source to sinks is critical for constraining the origin, transformation, and “hotspots” of contaminants in a river basin. To provide new spatiotemporal constraints on river chemistry, dissolved trace element concentrations were measured at 17 targeted locations across the Ramganga River catchment. River water samples were collected across three seasons: pre-monsoon, monsoon, and post-monsoon between 2019 and 2021. To remove the dependency of trace element concentrations on discharge, we used molar ratios, as discharge data on Indian transboundary rivers are not publicly available. The dataset reveals significant spatiotemporal variability in dissolved trace element concentrations of the Ramganga River. Samples collected upstream of Moradabad, a major industrial city in western Uttar Pradesh, are characterized by ~ 1.2–2.5 times higher average concentrations of most of the trace elements except Sc, V, Cr, Rb, and Pb, likely due to intense water–rock interactions in the headwaters. Such kind of enrichment in trace metal concentrations was also observed at sites downstream of large cities and industrial centers. However, such enrichment was not enough to bring a major change in the River Ganga chemistry, as the signals got diluted downstream of the Ramganga-Ganga confluence. The average river water composition of the Ramganga River was comparable to worldwide river water composition, albeit a few sites were characterized by very high concentrations of dissolved trace elements. Finally, we provide an outlook that calls for an assessment of stable non-traditional isotopes that are ideally suited to track the origin and transformation of elements such as Li, Mg, Ca, Ti, V, Cr, Fe, Ni, Cu, Zn, Sr, Ag, Cd, Sn, Pt, and Hg in Indian rivers.

**Supplementary Information:**

The online version contains supplementary material available at 10.1007/s10661-023-11665-0.

## Introduction

Water quality in most large river basins across the world has degraded because of intensified anthropogenic activities and population growth (Best, 2019; Häder et al., 2020; Immerzeel et al., 2010; Schwarzenbach et al., 2010). Monitoring the water quality of large river systems is therefore critical because they provide water and food security to 2.7 billion people (Best, 2019). Riverine water contaminants such as organic pollutants, pesticides, bacteria, antibiotics, and inorganic constituents such as heavy metals are routinely monitored (Boral et al., 2020; Gaillardet et al., 2014; Tiyasha et al., 2020). Monitoring river water quality to assess the impacts of rapid population and industrial growth in the Indian subcontinent has gathered momentum in the last few decades (Dwivedi et al., 2018; Elkiran et al., 2019; Islam et al., 2020; Jha et al., 2020; Khwaja et al., 2001).

In the Indian subcontinent, the Ganga—Brahmaputra basin extends over one million km^2^ and ranks among the most densely populated regions, with a population density of > 300 people/km^2^, and is home to over 0.6 billion people (Pandey et al., 2022). Previous studies have produced a rich body of information on the River Ganga water quality and how the riverine water quality has deteriorated over time (Amrutha & Warrier, 2020; Bindra et al., 2019; Boral et al., 2020; Kumar et al., 2020; Rani et al., 2011; Tripathi & Singal, 2019). As a result, site-specific monitoring of the water quality of the Ganga River has been extensively undertaken by India’s Central Pollution Control Board (CPCB) and Central Water Commission (CWC).

The efforts of CPCB and CWC clearly indicate that the most contaminated segment of the Ganga River begins at Kannauj (27.05° N, 79.91° E) and extends downstream to Varanasi (25.32° N, 82.97° E; CPCB, 2013; Narain, 2014; Tare et al., 2003). Previous studies show that the contaminants downstream of Kannauj are primarily derived from inputs from the Ramganga River, which is a major left-bank tributary of the River Ganga (Bowes et al., 2020; CPCB, 2013; Gurjar & Tare, 2019a, b; Narain, 2014; Sharmila & Arockiarani, 2016; Siddiqui et al., 2019). The Ramganga River is considered a highly polluted river as it is estimated to receive 235 MLD of industrial effluents and 227 MLD of domestic and municipal sewage via 11 tributaries and 4 major drains (CPCB, 2016). Further, the Ramganga catchment is exposed to extensive agricultural practices covering more than 60% of the land surface.

Nevertheless, the regulatory and academic research on water quality monitoring in the Ramganga basin primarily focuses on physicochemical and biological parameters, namely, temperature, pH, conductivity, alkalinity, total dissolved solids (TDS), total suspended solids (TSS), dissolved oxygen (DO), chemical oxygen demand (COD), biochemical oxygen demand (BOD), total coliforms (TC), various forms of nitrogen, and major ions (CPCB, 2011, 2013; Gurjar & Tare, 2019b; Khan et al., 2016, 2017, 2020; Pathak et al., 2018; Sarah et al., 2019; Sharma et al., 2003; Tare et al., 2003). Data on dissolved trace element concentration for the Ramganga River is sparse compared to physicochemical and biological parameters, despite the fact that dissolved trace elements are bioavailable (Arisekar et al., 2022; Tang et al., 2015; Taylor & Owens, 2009) and can pose serious health risks such as damage to nervous systems, brain cells, and other internal organs (Bosch et al., 2016; Cagnazzi et al., 2019; Salam et al., 2020; Tang et al., 2014).

This study, therefore, aims to provide a detailed understanding of how dissolved trace elements in the Ramganga River chemically evolve from their source to sink, their origins, and the possible causes behind these patterns. The three main objectives of this study are to (i) identify the “hotspots” of trace element contaminants and investigate the geochemical evolution of trace elements across the Ramganga Basin, (ii) investigate the current status of Ramganga River water quality and compare to other Indian and global rivers, and (iii) determine the role of natural versus anthropogenic factors controlling the dissolved trace element chemistry.

## Materials and methods

### Study area

The Ramganga River is the first major tributary to join the River Ganga near Kannauj District in the State of Uttar Pradesh after traveling ~ 600 km from its source in the Doodhatoli mountain ranges in the Himalayas (Singh & Sinha, 2022). The entire catchment of the river is fed mainly by the Indian Summer Monsoon (ISM), with limited contribution from snowmelt near its source (Asthana et al., 2015). The local climate across the basin varies significantly because of regional differences in the altitude levels ranging between > 3000 m above mean sea level (a.m.s.l.) at the headwaters to ~ 1000 a.m.s.l. in the downstream reaches within the Gangetic plain. Long-term meteorological observations (1969–2013) show that the average minimum and maximum temperature in the catchment area varies between 6 °C (January) to 36° (May–June) (Pathak et al., 2018). The average annual precipitation in the catchment is about 900 mm as per the 1996 to 2000 record (CWC, 2012).

**Fig. 1:**
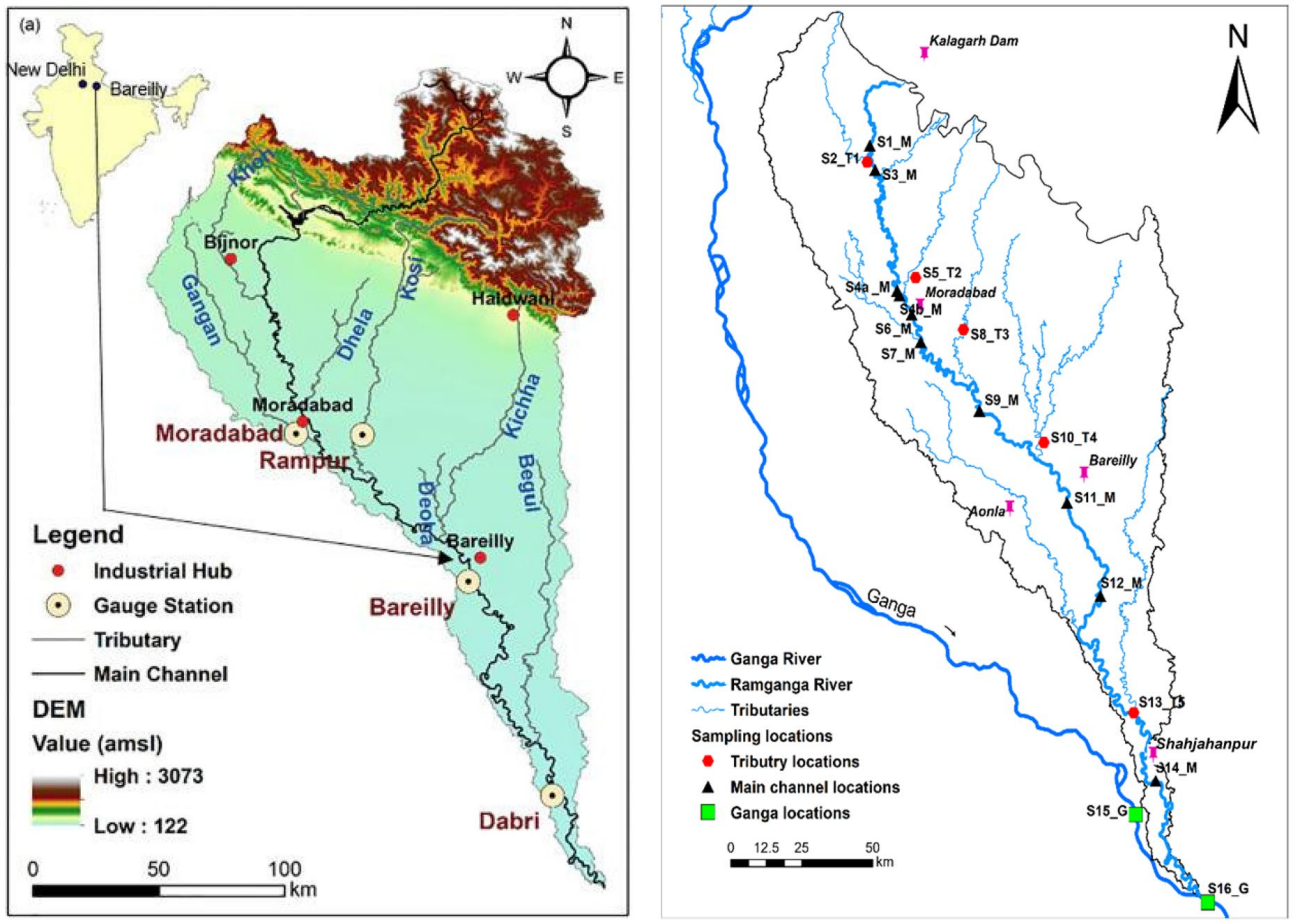
Map showing the Ramganga River and its tributaries and all the sampling locations. Major industrial hotspots (thumbtacks sym are also shown in the figure). Details of the sampling locations can be found in Table S1

The lithology of the basin comprises shale, slate, quartzite, mica schist, granite, and quarzitic rocks in the upper reaches, while the downstream region is characterized by Gangetic alluvium consisting of loamy and clayey-loamy soils (Gupta & Joshi, 1990; Panwar et al., 2020). More than half of the basin's total area is used for agricultural cultivation, whereas about 20% of the area is covered by grassland, barren land, and urban centers (Pathak, 2017). Upon exiting the hilly terrains of the Himalayan foothills, the river drains through densely populated industrial areas, including Bijnor, Moradabad, Rampur, and Bareilly Districts of Uttar Pradesh (Fig. 1). The Ramganga River provides 17.2 billion cubic meters of discharge, which is equivalent to approximately 3% of the total flow of the Ganga.

### River water sampling

Surface water samples (*n* = 81) were collected from ten sampling sites located along the ~ 400 km stretch of the Ramganga River. The upstream sampling point was ~ 50 km downstream of the Kalagarh Dam (29° 31 ′N 78° 45 ′E), and the last point was upstream of the Ramganga-Ganga River confluence near the township of Kannauj. Samples were also collected from the tributaries joining the Ramganga River and two locations on the Ganga River, upstream and downstream of the Ramganga-Ganga confluence (Fig. 1 and Supplementary Table S1). The distance between two consecutive sampling stations ranges between 5 and 40 km. These sampling sites were strategically chosen to capture the upstream and downstream locations of major industrial hubs and urban centers. The sampling campaigns were carried out during the monsoon season (August–September) and post-monsoon (November–December) seasons corresponding to 2019 and 2020. For the pre-monsoon season (March), only one fieldwork campaign (March 2020) was carried out due to the COVID-19 pandemic-related restrictions.

Approximately 2 L of water were collected in pre-cleaned high‐density polyethylene (HDPE) bottles, and a subsample was filtered through 0.22 μm Millipore polyethersulfone (PES) membranes into 125 mL pre-cleaned HDPE bottles. The filtered subsamples were acidified to 2% (v/v) ultrapure HNO_3_ for trace metal analysis. The constituents that passed through the 0.22 μm PES membranes are termed here as “dissolved fractions” that include both the truly dissolved fractions (< 0.001 μm) and the colloidal fractions (0.001–0.22 μm). We would like to mention that we did not proceed with ultrafiltration techniques as our study did not aim to study the speciation, solubility, toxicity, or bioavailability of the dissolved trace constituents.

All bottles were rinsed three times with filtered river water from the respective sites and stored at 4 °C until analysis. Physicochemical parameters (temperature, electrical conductivity (EC), and pH) were measured immediately in the field at each sampling location using a portable multiparameter sonde (YSI Professional Plus). Detailed sampling analytical protocols have been outlined by Boral et al. (2020) and Shukla et al. (2021).

### Trace elemental analysis

Dissolved inorganic constituent concentrations were analyzed for the following trace elements: lithium (Li), aluminum (Al), scandium (Sc), vanadium (V), chromium (Cr), cobalt (Co), nickel (Ni), copper (Cu), arsenic (As), selenium (Se), rubidium (Rb), strontium (Sr), molybdenum (Mo), barium (Ba), lead (Pb), and uranium (U). Trace element concentrations were measured using an Agilent 8900 triple quadrupole inductively coupled plasma mass spectrometer (QQQ-ICP-MS) at the Department of Earth Sciences, Indian Institute of Technology Kanpur. Briefly, 6 mL solution of the water samples, blanks (i.e., deionized water acidified to 5% with ultrapure HNO_3_), and a reference standard NIST 1643f (Trace Elements in Water, National Institute of Standards and Technology) were spiked with 5 ppb rhodium (Rh) solution.

The procedural blank was quantified by analyzing the blank samples. NIST 1643-f standard was analyzed for data quality assurance, and a 5 ppb Rh solution was used as an internal standard to correct for any signal drift during the course of the analysis. Elemental concentrations were measured against a 7-point calibration curve (*R* ≥ 0.997). The final concentrations were blank–corrected using the average procedural blank concentrations, and the instrument drift and matrix effect was corrected by Rh normalization. The data quality and reproducibility of the analysis were continuously monitored by periodically running reference standard NIST 1643f and calibration standards (for Sc and U) (Table S2). Fifteen replicates were measured to monitor the reproducibility of the analysis, and the results showed an average reproducibility of 102 ± 4% (*n* = 15, 1 SD). The overall accuracy was < 8%, with a precision of ≤ 3%. The analysis results of all the samples are provided in Supplementary Table S3.

### Chemometric assessments of dissolved load

Univariate analysis was performed on dissolved trace element datasets to determine the range, mean, and standard deviation. To compare the trace element and heavy metal concentrations among various sampling sites in different monsoon seasons, the geochemical results were subjected to bivariate analyses, including Pearson’s correlation analysis and one‐way analysis of variance (ANOVA) at significance *p* ≤ 0.05. A post hoc Tukey’s test was also performed to do a pairwise comparison of the different sampling sites. The robustness of the ANOVA results was checked with Welch tests and Brown‐Forsythe tests. Grubb’s test was performed to remove the significant outliers in the data set prior to any statistical analyses.

## Results and discussion

### Spatial evolution of river chemistry

Statistics of the dissolved trace element concentrations in water samples collected from the Ramganga mainstream (hereafter referred to as “RG-MS”), Ramganga tributaries (hereafter referred to as “RG-T”), and Ganga River are reported in Table 1, whereas individual data points can be found in Supplementary Table S3. The concentrations of alkali (Li and Rb) and alkaline (Sr and Ba) metals, as well as Sc, Al, As, and U, at individual sites exhibit significant variability (Figs. S1-S2). For instance, concentration of Sr ranges from 62 to 318 µg L^−1^, with the maximum value recorded at the Dhela tributary. The Dhela tributary site (S5-T2) is also characterized by higher concentrations of other ions such as Li, As, Sc, Rb, and heavy metals. The heavy metal concentrations also exhibit up to two orders of magnitude variability in the RG-MS. In contrast the samples from RG-T and Ganga exhibit only one order of magnitude variability in concentration. Downstream sites from Moradabad namely S6-M and S7-M, typically exhibit higher concentrations of dissolved trace elements. Trace element concentration “hotspots” were observed near urban centers such as the cities of Shahjahanpur (S11-M) and Bareilly (S14-M).

**Table 1:** Statistical summary of dissolved trace element concentrations (in µgL^−1^) measured in water samples collected from the main stem of RG-MS, its tributaries (RG-T), and Ganga River. The constituents that passed through the 0.22 μm PES membranes are termed here as “dissolved.” SRM 1643f (Trace Elements in Water, National Institute of Standards and Technology) was analyzed to assess the data quality. Individual data points can be found in Supplementary Table S3. Global average river and Ganga River water composition are from Viers et al. (2009) and Boral et al., 2020, respectively. The drinking water standard is from the national and international standards (BIS, 2012; WHO, 2011). Blank cells reflect that the concentrations are below the detection limit. Min. refers to the minimum, max. refers to the maximum, *n* refers to the number of analyses, and 1σ refers to 1 standard deviation of the data

	**Ramganga main stream (*n* = 46)**				**Ramganga tributary (*n* = 25)**				**Ganga River (*n* = 10)**				**Global**	**Drinking**	**Ganga**
	Min.	Max.	Avg.	1σ	Min.	Max.	Avg.	1σ	Min.	Max.	Avg.	1σ	River	Water	River
**Li**	0.81	4.88	2.31	1.20	0.75	4.95	2.67	1.24	2.06	3.84	2.99	0.55	1.84		6.20
**Al**		16.2	5.01	4.37		15.0	4.71	3.89		41.9	14.5	12.0	32.0	200	16.5
**Sc**		0.93	0.37	0.30		1.41	0.42	0.41		0.43	0.27	0.12	1.20		0.33
**V**	0.01	3.91	1.66	1.05	0.52	5.28	1.93	1.37	0.83	2.02	1.29	0.36	0.71		10.1
**Cr**	0.003	0.97	0.23	0.25	0.01	0.59	0.18	0.17	0.03	0.28	0.14	0.09	0.70	50	1.00
**Co**	0.02	0.40	0.08	0.06	0.01	0.34	0.10	0.09	0.01	0.05	0.02	0.01	0.15		0.30
**Ni**	0.08	8.70	1.93	2.13	0.08	1.65	0.57	0.42	0.08	0.37	0.24	0.09	0.80	70	2.80
**Cu**	0.37	11.7	2.86	2.64	0.23	2.12	1.05	0.54	0.82	2.60	1.51	0.70	1.48	2000	3.30
**Zn**	0.07	31.7	4.53	7.81	0.08	4.62	1.20	1.08	0.31	2.41	0.96	0.63	0.60	1500	1.12
**As**	1.52	7.47	3.77	1.40	0.17	11.7	4.29	2.85	2.45	6.25	4.77	1.14	0.62	10	9.00
**Se**		1.44	0.47	0.37		1.31	0.44	0.28		0.54	0.27	0.20		40	0.35
**Rb**	0.68	10.2	3.61	2.73	0.63	5.39	1.95	1.15	2.15	3.34	2.70	0.37	1.63		5.20
**Sr**	71.1	278	180	53.1	62.1	318	194	80.0	59.8	117	88.9	21.4	60.0	4000	2689
**Mo**		2.50	0.95	0.59		3.22	1.37	0.92		1.74	1.03	0.42	0.42		0.78
**Ba**	27.6	101	62.6	17.9	25.2	134	53.6	23.1	27.9	49.3	37.6	6.72	23.0	700	73.3
**Pb**		0.42	0.11	0.11		0.13	0.06	0.04		0.09	0.05	0.02	0.08	10	0.40
**U**	0.24	2.40	1.24	0.65	0.21	3.11	1.36	0.69	1.51	4.63	2.67	0.98	0.37	30	2.79

**Fig. 2:**
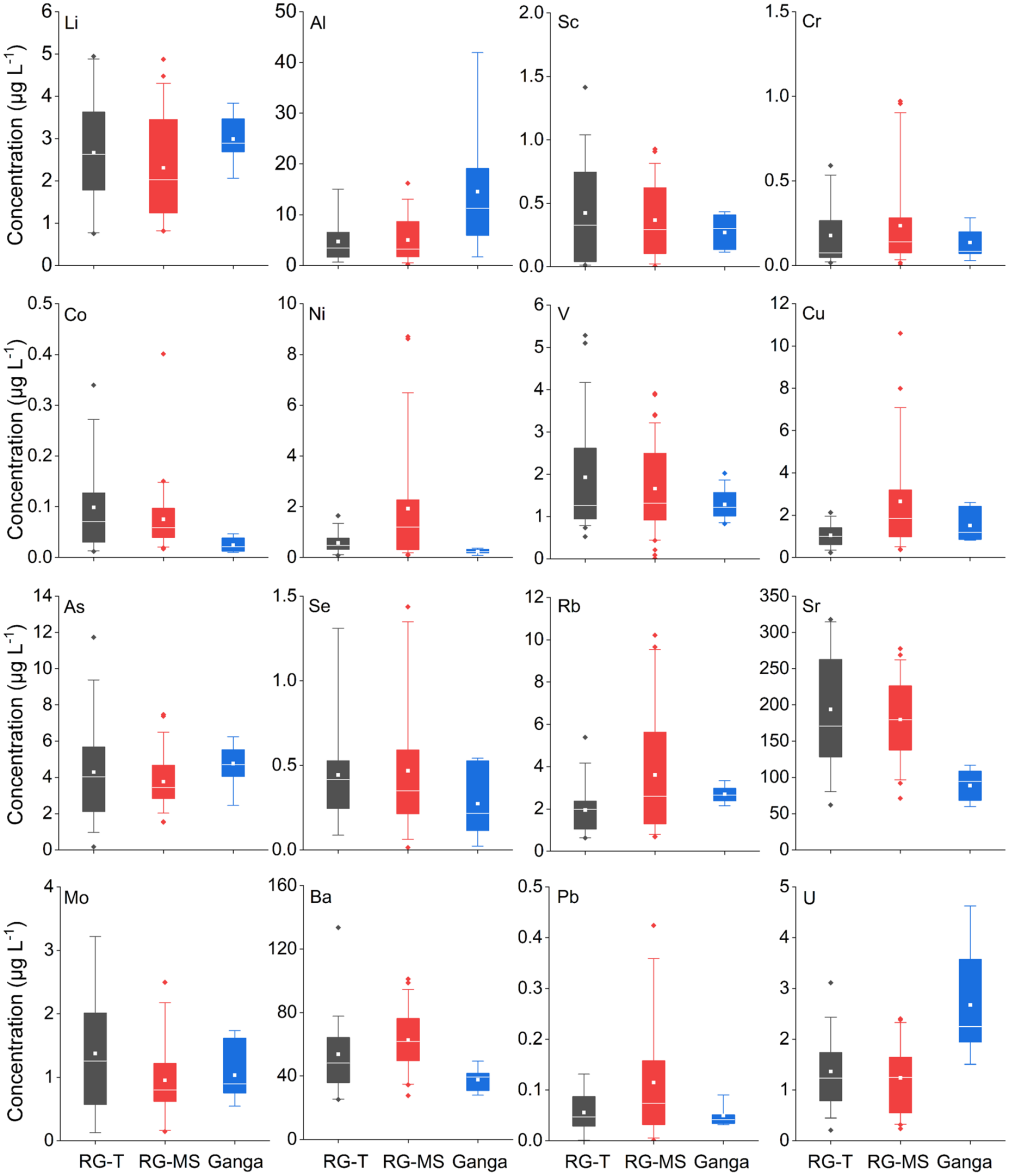
Dissolved average geogenic trace element concentrations in the RG-T, RG-MS, and the Ganga River. Box plots represent the interquartile range (box), mean (open square inside the box), median (line inside the box), and 5^th^ and 95^th^ percentiles (whiskers) for each fraction

Like individual sites, the mean annual concentration of the trace elements display significant yet non-systematic variability in both the tributaries and rivers (Fig. 2). For instance, Li abundance follows the order of Ganga River > RG-MS > RG-T, whereas the order for V is RG-T > RG-MS > Ganga River. Among the trace elements, Cr, Co, Se, and Pb exhibited lowest mean annaul concentration (< 0.5 μg L^−1^), whereas Ba and Sr were the most abundant, with concentrations exceeding 30 μg L^−1^. One-way ANOVA analysis performed for mean comparison reveals that Al, Co, Ni, Cu, Rb, Sr, Ba, and U were significantly different (at *p* ≤ 0.05) between RG-MS, RM-T, and Ganga River. There are only a few sites in the main stem of the Ramganga River that contains a higher abundance of dissolved trace constituents. In most cases, enrichment and depletion trends observed in the main stem of the Ramganga river are controlled by the tributary contributions. The sampling point downstream of the Ramganga-Ganga confluence point shows that the dissolved inorganic constituent gets diluted in the Ganga River.

The average concentrations of heavy metals in the RG-MS are lower than most of the existing datasets of the Ganga River and its tributaries. This difference arises because previous studies were primarily focused on identiying contamination “hotspots” around large industries and urban centers, while our aim was to capture the entire variability from the source to sink. Likewise, the annual averages of most dissolved trace elements in both tributaries and the mainstream in the present study is 1–2 orders of magnitude lower than the water composition reported for the year 2014–2016 (Gurjar & Tare, 2019a). A recent study revealed that the high dissolved metal load in the RG-MS can be primarily attributed to the increased metal content in tributaries that transport domestic and industrial waste (Khan et al., 2020). However, this is not the case in the present study, as the concentrations of the dissolved trace metal in the mainstream are similar to or lower than its tributaries, probably due to the cessation of the industrial and anthropogenic activities during the COVID pandemic lockdown imposed from 24^th^ March–31^st^ May 2020. Therefore, this provides indirect evidence that anthropogenic activities in the Ramganga basin are primarily responsible for pollution observed under the “business as usual” scenario. These anthropogenic activities must be strongly regulated in order to improve the water quality and mitigate the further deterioration of the Ramganga River.

The average concentrations of trace metals in the RGMS are similar to or higher than some of the major rivers of the world (Supplementary Table S4). Similarly, most of the trace element concentrations in the RGMS exhibit a closer resemblance with the Kosi river due to limited anthropogenic perturbance since samples were collected during the COVID pandemic lockdown (Boral et al., 2020). Under certain circumstances, natural factors such as interactions with freshly ground rock flour due to intense physical weathering in the mountainous headwaters may also lead to elevated metal concentrations, as observed in the Gandaki river of central Nepal (Pant et al., 2020). In contrast, dissolved trace element load reported in major rivers viz. Yamuna, Gomti, and Ganga downstream from Farrukhabad to Manikchak (Boral et al., 2020; Iqbal et al., 2019), Yarlung Tsangpo, Yangtze, and Indus (Qu et al., 2014; Wu et al., 2009), Mississippi (Reiman et al., 2018), Thame (Neal et al., 2006), and Ghana Stream (Asante et al., 2007) is significantly higher than RGMS owing to the greater extent of human interventions or manifestation of anthropogenic activities during the sampling campaign.

### Evolution of river chemistry in time

**Fig. 3:**
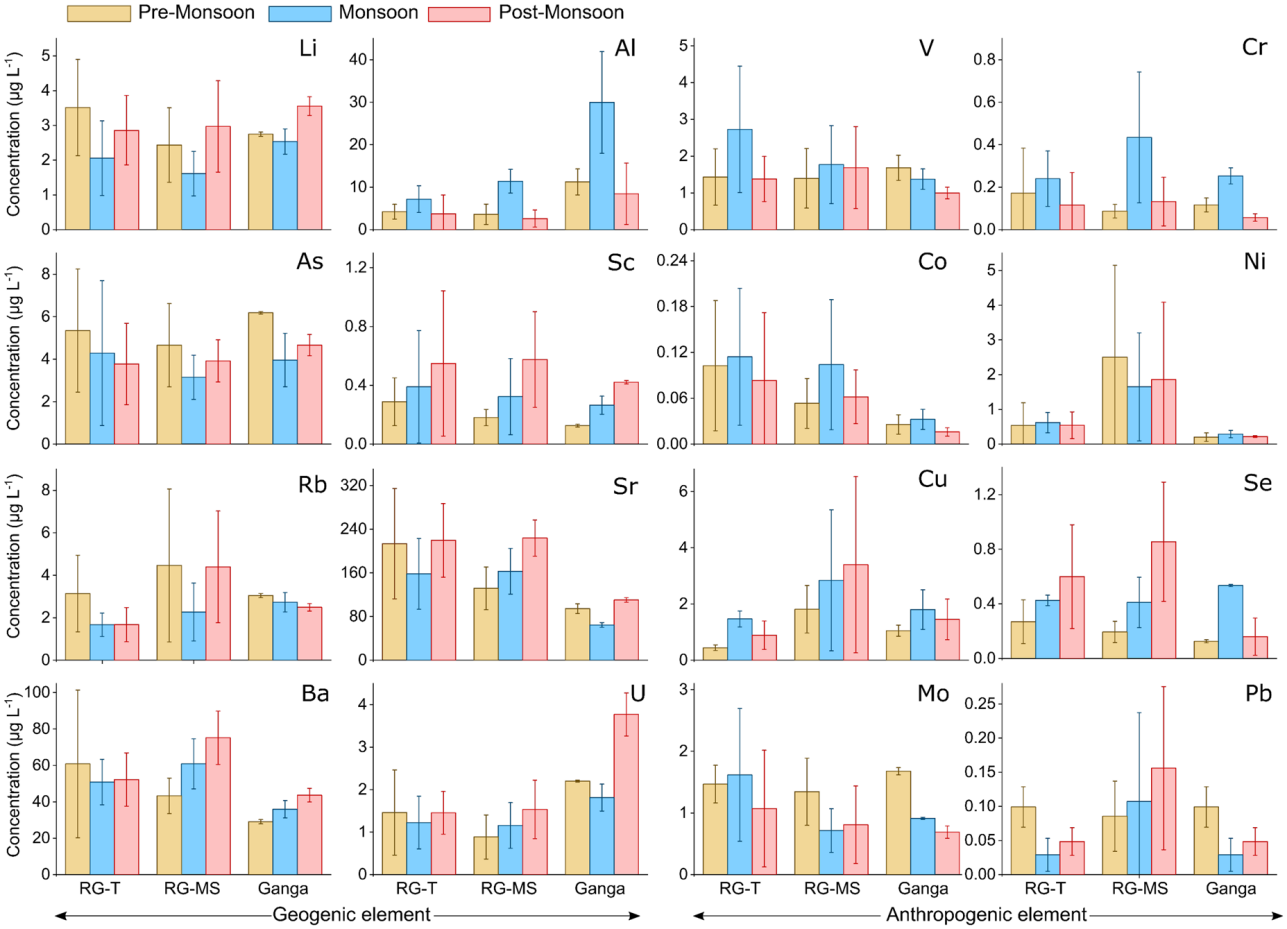
Average seasonal trace element concentrations in the RG-T, RG-MS, and the Ganga River. The error bar represents one standard deviation in the dataset

The Ganga and its tributaries, including the Ramganga River, have been extensively studied for decades (Gurjar & Tare, 2019a, b; Khan & Tian, 2018; Khan et al., 2016; Panwar et al., 2017, 2020; Pathak et al., 2018; Tare et al., 2020). These studies—the only exception being the Gurjar and Tare (2019b) study—are either based on remote sensing or mostly focused on specific sites that are close to industrial centers. Moreover, these studies did not capture the seasonal variability of the river, which is critical considering that water discharge changes substantially across the seasons due to the monsoon climate. For example, satellite microwave radiometry-based discharge data upstream of the confluence between the Ramganga and Ganga Rivers reveal variations in discharge ranging from 200 to 2,000 m^3^/s (Brakenridge et al., 2022). This study shows the average dissolved inorganic constituent concentrations in RG-MS exhibit large temporal variability (Fig. 3 and Table 2). ANOVA results indicate that seasonal mean concentrations are significantly different (at *p* ≤ 0.05), except for V, Co, Ni, Cu, and Pb. We did not find any specific differences in patterns between pre-monsoon and post-monsoon seasons (Table 2). Figure 3 shows that the RG-MS had the least enrichment of alkali metals (Li and Rb) in the monsoon season, indicating a dilution trend due to rainfall contributions from the Indian Summer Monsoon. Conversely, heavy metals like Cr and V show the most enrichment in the monsoon season, probably indicating the flushing of catchment pollutants by rainfall runoff. There exists a significant seasonal variability in the dissolved trace element concentrations in RG-T (Table 2).

**Table 2:** Statistical summary of dissolved trace element concentrations (in µgL^−1^) across the three seasons: pre-monsoon (April–June), monsoon (July–September), and post-monsoon (October–December) over three years (2019–2021). Individual data points can be found in Supplementary Table S3. Min. refers to a minimum, max. refers to the maximum, *n* refers to the number of analyses, and 1σ refers to 1 standard deviation of the data

Element	**Ramganga main stem (RG-MS)**						**Ramganga tributary (RG-T)**						**Ganga**					
	Pre-monsoon (*n* = 10)		Monsoon (*n* = 18)		Post-monsoon (*n* = 18)		Pre-monsoon (*n* = 5)		Monsoon (*n* = 10)		Post-monsoon (*n* = 10)		Pre-monsoon (*n* = 2)		Monsoon (*n* = 4)		Post-monsoon (*n* = 4)	
	Avg.	1σ	Avg.	1σ	Avg.	1σ	Avg.	1σ	Avg.	1σ	Avg.	1σ	Avg	1σ	Avg	1σ	Avg	1σ
Li	2.44	1.07	1.61	0.64	2.97	1.31	3.51	1.39	2.06	1.08	2.86	0.99	2.75	0.06	2.54	0.36	3.56	0.27
Al	3.60	2.40	11.4	2.79	2.59	2.00	4.22	1.75	7.17	3.15	3.71	4.44	11.24	3.06	29.9	12.0	8.44	7.22
Sc	0.18	0.06	0.32	0.26	0.58	0.33	0.29	0.16	0.39	0.38	0.55	0.49	0.13	0.01	0.26	0.06	0.42	0.01
V	1.39	0.81	1.77	1.06	1.69	1.12	1.43	0.77	2.73	1.72	1.38	0.61	1.68	0.34	1.37	0.28	1.00	0.16
Cr	0.09	0.03	0.43	0.31	0.13	0.11	0.17	0.21	0.24	0.13	0.12	0.15	0.12	0.03	0.25	0.04	0.06	0.02
Co	0.05	0.03	0.10	0.09	0.06	0.04	0.10	0.09	0.11	0.09	0.08	0.09	0.03	0.01	0.03	0.01	0.02	0.01
Ni	2.50	2.64	1.65	1.55	1.86	2.23	0.54	0.65	0.62	0.29	0.54	0.39	0.20	0.12	0.29	0.11	0.22	0.02
Cu	1.81	0.85	2.84	2.51	3.40	3.14	0.44	0.10	1.47	0.28	0.89	0.51	1.05	0.20	1.80	0.70	1.45	0.72
Zn	2.50	2.13	3.87	7.64	6.34	9.46	1.57	1.63	1.46	0.88	0.72	0.49	0.49	0.12	0.83	0.44	1.41	0.71
As	4.66	1.96	3.14	1.04	3.92	0.99	5.35	2.90	4.28	3.41	3.77	1.92	6.19	0.06	3.96	1.26	4.66	0.50
Se	0.19	0.08	0.41	0.18	0.85	0.44	0.27	0.16	0.43	0.04	0.60	0.38	0.13	0.01	0.53	0.01	0.16	0.14
Rb	4.47	3.60	2.27	1.36	4.40	2.63	3.14	1.80	1.67	0.55	1.67	0.81	3.05	0.10	2.73	0.46	2.49	0.18
Sr	132	39.1	163	41.9	224	33.1	213	101	158	64.8	220	67.5	94.4	8.88	64.6	4.22	110	3.99
Mo	1.34	0.54	0.71	0.35	0.81	0.63	1.47	0.31	1.62	1.08	1.07	0.95	1.68	0.06	0.91	0.02	0.69	0.10
Ba	43.3	9.75	60.9	13.8	75.2	14.7	60.83	40.55	50.89	12.52	52.2	14.6	29.1	1.18	35.9	4.75	43.6	3.76
Pb	0.09	0.05	0.11	0.13	0.16	0.12	0.10	0.03	0.03	0.02	0.05	0.02	0.04	0.00	0.04	0.01	0.09	0.00
U	0.88	0.52	1.16	0.54	1.54	0.69	1.46	1.00	1.22	0.62	1.45	0.50	2.20	0.02	1.81	0.32	3.77	0.51

The individual sites also demonstrate substantial variability. Site-specific seasonal concentration revealed higher geogenic elements during pre- and post-monsoon seasons, as reflected in the average datasets, due to the expected lower discharge. The variability in elemental concentrations between monsoon and non-monsoon seasons becomes higher downstream of major urban centers. For example, upstream of Moradabad city (S4a-M), variability in the concentration of geogenic elements between monsoon and non-monsoon seasons is lower, but the variability increases downstream of Moradabad (S6-M) and Bareilly (S12-M). Understanding the temporal variability downstream of major cities such as Moradabad is very complex due to the significant modulation of the signals by anthropogenic source inputs (Khan et al., 2022; Sarah et al., 2019).

It is noteworthy to mention that in upstream samples up to S4a-M, i.e., prior to the industrial region (Moradabad), some of the trace elements, such as Sr, As, and Ba, exhibit pronounced temporal variability and enrichment (Supplementary Fig. S1). The concentration in this region is primarily controlled by the combined effect of varying extent of rock/sediment weathering processes (Gaillardet et al., 2014; Pant et al., 2020). The higher concentrations in these pristine sites (site upstream of S4a-M) are likely due to the interaction of water with freshly weathered material transported from the headwater regions (Tranter & Wadham, 2014).

**Fig. 4:**
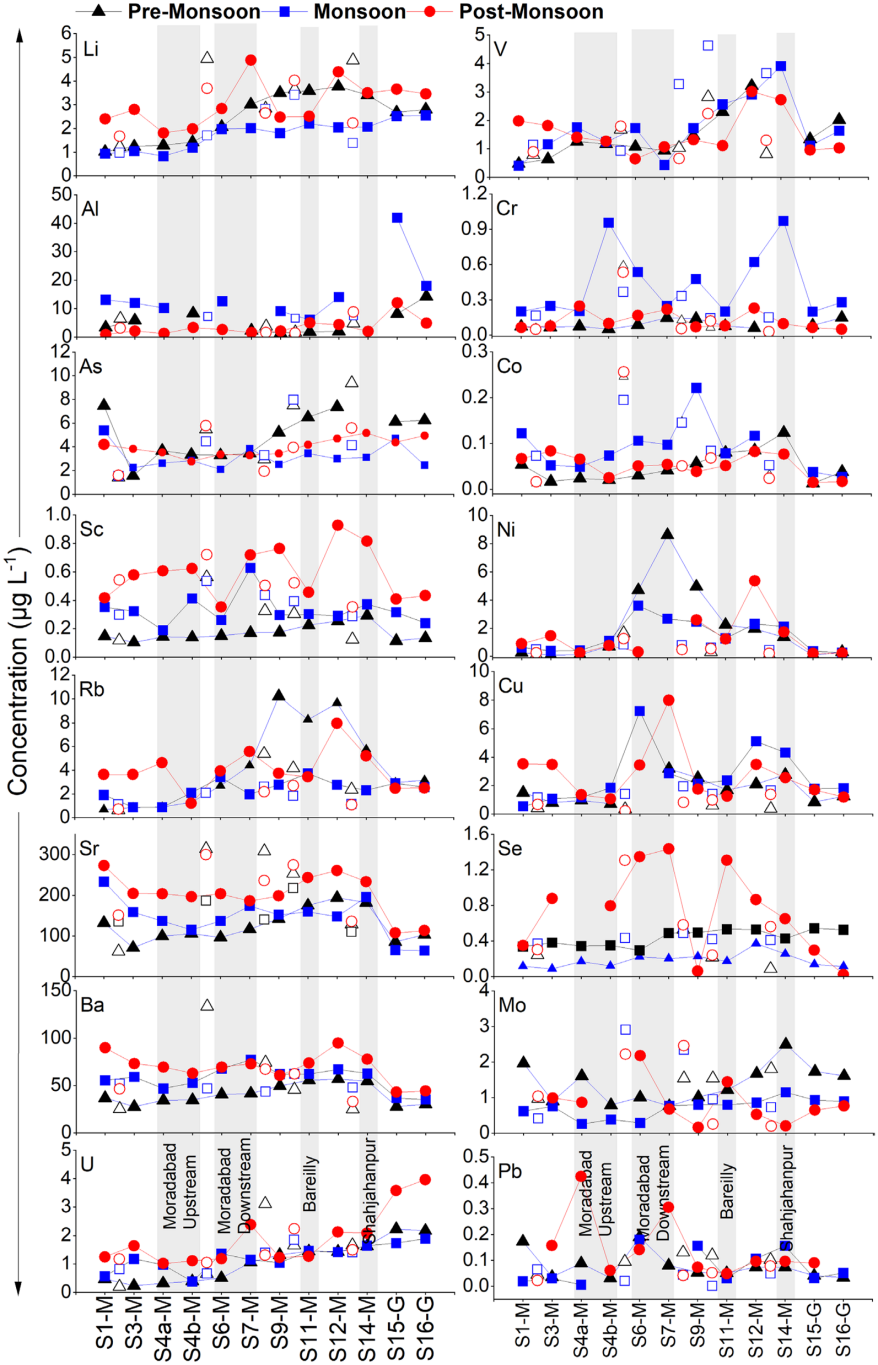
Spatiotemporal variability of dissolved trace element concentrations in the RG-MS and its tributaries (RG-T: open circle) and the Ganga River

The temporal variability of the dissolved trace elements in RG-MS is consistent with previous findings (Gurjar & Tare, 2019b), Ganga (Boral et al., 2020), and Gandaki River (Pant et al., 2020) of Nepal. Further, most of the trace element concentrations are comparable to the global average river composition, the Ganga River, and drinking water guidelines prescribed by WHO and Indian standards (Fig. 4). This suggests that the water quality composition of the Ramganga is comparable with the regulatory values and is found not to be grossly polluted with trace elements during the sampling period. This is probably due to the reduction in industrial pollution following the nationwide COVID restrictions in India. Therefore, we emphasize that the probability of dissolved trace element contamination is high under more typical circumstances, and the overall river water composition of the Ramganga during the sampling campaign is comparable to or lower than the global average, including the Ganga River. However, the river water quality in the future might degrade with the activation of industrial and anthropogenic activities, in line with earlier studies.

### Controls on dissolved trace metal concentration of the Ramganga Basin

**Fig. 5:**
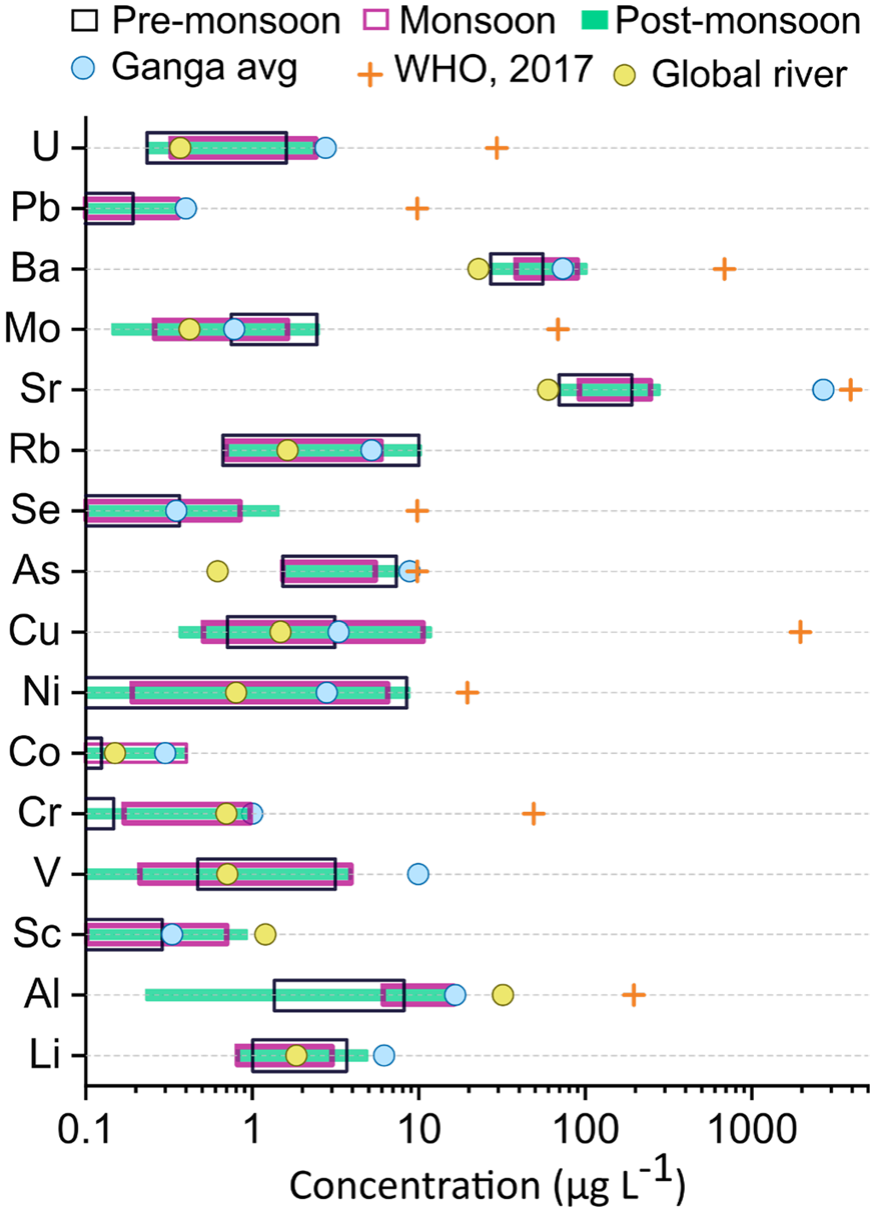
Overview of the seasonal dissolved trace element concentrations in the mainstream of the Ramganga River compared to the average trace element composition of the Ganga River (Boral et al., 2020), global average river water composition (GR), and World Health Organization drinking water standards (WHO Std.) is from Viers et al. (2009) and WHO (2011), respectively

**Fig. 6:**
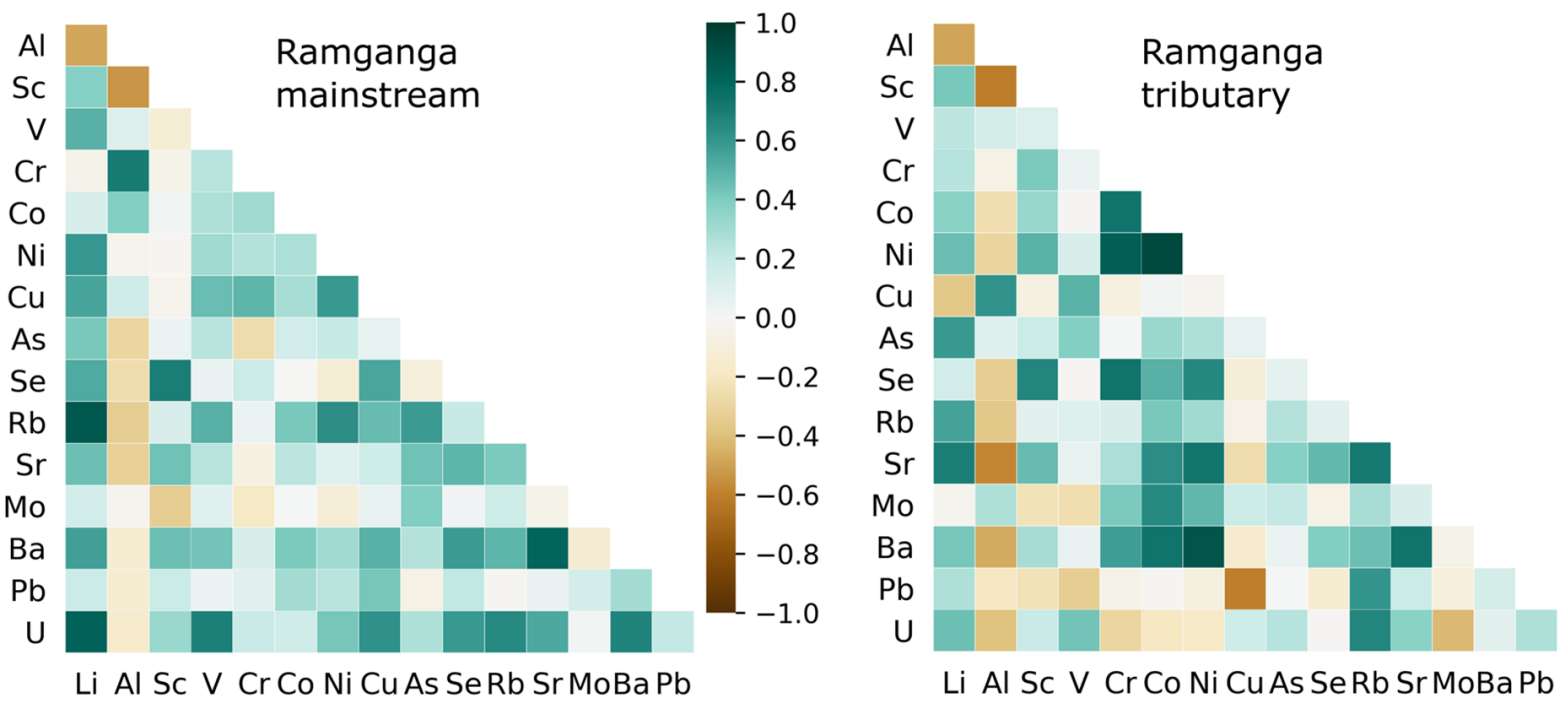
Correlation heat maps showing the relationships of trace elements in the RG-T and RG-MS. Green shade indicates positive relationship, whereas brown sheds represent negative relationships

The dissolved inorganic constituents of the Ramganga River show large spatiotemporal variability (Figs. 3 and 5). This variability can be controlled by both geogenic and anthropogenic factors. Geogenic factors contributing to the dissolved geochemical load of a river mostly include the intensity of water–rock interaction in the river catchment (Tranter & Wadham, 2014; Xu et al., 2020). Anthropogenic sources can vary widely and include runoff from urban sites, agricultural fields, mine drainage, as well as industrial and municipal effluents (Best, 2019). In order to gain a deeper insight into the factors governing the occurrence, distribution, and sources of the trace elements in the Ramganga basin, we studied the relationship of trace elements with each other (Fig. 6).

Given that the geochemical behavior of trace elements is interrelated (Gaillardet et al., 2014), Pearson correlation analysis was performed to study the correlations among the elements (Fig. 6). In the RG-MS, alkali and alkaline metals, as well as uranium, exhibit moderate to strong positive correlation among themselves. This implies a probable geogenic origin as alkali and alkaline metals elements are predominantly sourced from rocks. Similar intra-relations among geogenic elements are observed in the RG-T. However, in the case of RG-T, we observed a stronger positive inter-correlation between geogenic elements and heavy metals, specifically, Ni, Co, Cr, Mo, and Se. This indicates that the heavy metals in RG-T most likely have a geogenic origin, and the contributions of industrial and municipal effluents were low (Gaillardet et al., 2014; Shukla et al., 2021). It is worth mentioning that the trace elements in the Ganga River do not correlate with each other, as previously observed (Boral et al., 2020; Gurjar & Tare, 2019b; Khan et al., 2020, 2022, 2017), and therefore, the anthropogenic sources of these elements provide a significant proportion of the load.

**Fig. 7:**
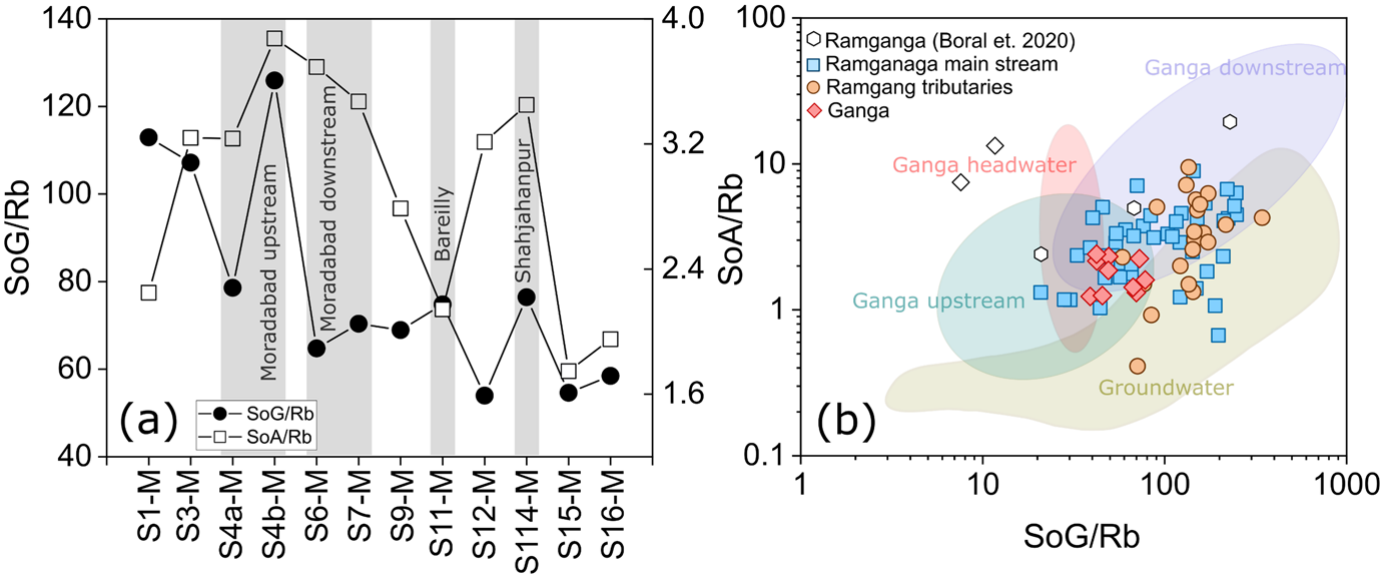
**a** Spatial variability of trace metals illustrated through the Rb-normalized molar sum of geogenic elements (SoG: ∑Li, Sr, Ba/Rb) and anthropogenic elements (SoA: ∑V, Cr, Ni, Co, Pb/Rb) in the RG-T and RG-MS. **(b)** Comparison of the data in the Ramganga River with river and groundwater samples (Boral et al., 2020)

Since the concentration of dissolved riverine trace elements depend on water discharge, we use molar ratios to remove the dependency of trace element concentrations on discharge to evaluate the predominant source of trace elements. We use elements such as Li, Rb, Sr, and Sc for normalization as these elements have very few industrial applications and can be used as a proxy for natural/geogenic sources (Boral et al., 2020). Heavy metals such as Cr, V, Co, Ni, Cu, and Pb often exhibit higher concentrations in dissolved load due to their limited mobility during weathering and are mostly due to anthropogenic inputs (Gaillardet et al., 2014). Furthermore, it has been observed that inferring sources based on the variabilty of individual trace elements may be biased and challenging since their abundances are controlled by a complex mixture of various sources. Therefore, to understand the sources, we used groups of elements rather than individual elements. Figure 7a illustrates the covariation of the Rb normalized sum of predominantly geogenic elements: (SoG: ∑ Li, Sr, Ba/Rb) versus the sum of predominantly anthropogenic elements (SoA: ∑V, Cr, Ni, Co, Pb/Rb) in the Ramganga Basin. In upstream samples, both the SoG and SoA exhibited higher concentrations because of the higher intensity of chemical weathering in the headwaters. A strong positive correlation (*R* = 0.75) between Rb-normalized SoG and SoA at the initial four sampling sites and the clustering of samples in the headwater fields further supports the predominant role of geogenic (weathering) processes in dissolved trace elemental enrichment at these sites. The sum of the predominantly anthropogenic elements surpassed the sum of the predominantly geogenic elements at the second sampling station, most likely due to the contribution from the industrialized Dhela tributary (S5-T2)—one of the most anthropogenically-polluted tributaries of the Ramganga River. Similarly, at several downstream locations, such as near Moradabad (S4b-M), Bareilly (S11-M), and Shahjahanpur (S14-M), the sum of the predominantly anthropogenic elements was significantly higher compared to the rest of the catchment. Since all the ratios were normalized with Rb, and hence, we removed any discharge dependency on the concentration. Figure 7b clearly shows that RG-MS is significantly enriched compared to the Ganga River downstream of the Ramganga confluence point.

However, the dissolved inorganic constituents discharged by the Ramganga gets diluted in the Ganga River as evidenced by a lower SoG: ∑ Li, Sr Ba/Rb, and SoA: ∑V, Cr, Ni, Co, Pb/Rb in the Ganga River sampled downstream of the Ramganga-Ganga River confluence point (Fig. 7b). Figure 7 also shows that the dissolved trace element molar ratios of the Ramganga basin and the entire Ganga basin from source to sink (Fig. 7b) are primarily controlled by a complex interplay between the varying extent of anthropogenic and natural activities as well as rain/surface water contributions.

### Limitation of this study and outlook

The major limitation of this study is the unavailability of discharge data considered classified information by the Government of India. Although satellite microwave radiometry-based discharge data is available from River and Reservoir Watch version 4.5 using the database of the Dartmouth Flood Observatory (Brakenridge et al., 2022), it is only available at one site (28.079° N, 79.47° E), which is upstream of the confluence between the Ramganga and Ganga Rivers, and did not serve our purpose as we sampled the entire river catchment. The unavailability of the discharge data means that pollution loads cannot be calculated and severely limits our capability to determine the role of discharge in observed spatiotemporal variability as we were unable to calculate the concentration (C) − discharge (Q) relationships using the log(C) versus log(Q) linear relationships (Baronas et al., 2017; Godsey et al., 2009). As an alternative option, we used molar ratios to remove the dependency of trace element concentrations on discharge to evaluate the predominant source and spatiotemporal variability of trace elements. Further, an ideal process to track sources of trace elements would require the characterization of all possible source end-members spanning from industrial effluents, domestic sludge, and agricultural field runoffs that contribute to the pollution load of the Ramganga. However, this is a challenging task as it is estimated that the total wastewater discharge directly or indirectly into the river Ramganga is nearly 235 MLD from various different industrial sectors (CPCB, 2016). Therefore, identifying individual point-source pollution sources for each element will be a complex task.

Finally, we would like to emphasize the use of stable isotopes as source indicators. In the last 15 years, a range of new isotopic tools—e.g., the non-traditional isotopes such as, e.g., Li, Mg, Ca, Ti, V, Cr, Fe, Ni, Cu, Zn, Sr, Ag, Cd, Sn, Pt, and Hg—have emerged largely due to the innovation of the Multi Collector Inductively Coupled Plasma Mass Spectrometer (MC-ICPMS) which are ideally suited to track the evolution and source of each individual element. Stable isotope ratios are excellent complements to dissolved concentrations and elemental ratios because they can only be sensitive to processes and sources that change the overall budget of the element studied. The use of non-traditional stable isotopes together with element concentration, ratios, and routinely used isotopic tracers such as O, H, Sr, and others can present a new avenue of research that has great potential to enhance our understanding of the sources and fates of chemical pollutants in Indian rivers. For example, Cd isotopes [δ^114/110^Cd = [(^114/110^Cd)_sample_/(^114/110^Cd)_standard_ – 1]*1000] of river water were used to track and quantify the contribution of acid mine drainage in a river in southern China (Yang et al., 2019). Similarly, Zn isotope (δ^66/64^Zn) compositions of river water were used to trace metal sources at a catchment scale (Desaulty et al., 2020). The availability of this new isotopic systematics has brought in a paradigm shift in the understanding of trace metal dynamics in riverine catchments. However, to the best of our knowledge, the non-traditional stable isotope data signature of Indian rivers is unknown. Such data would not only help quantify the source of metal contaminants in rivers but it would also help to better understand their evolution and the processes that control their abundance from their source to sink. Apart from identifying the sources and processes, quantitative knowledge about each contaminant will also help mitigate important river contamination problems.

## Conclusions

The 2-year long seasonal sampling of River Ramganga and its tributaries reveals that the dissolved inorganic constituents of the rivers show large variability in space and time. Further, the basin-wide characterization of dissolved inorganic species presented in this study shows that:

- The pristine upstream locations above large cities are characterized by the enrichment of dissolved inorganic species—mostly geogenic elements—due to intense water–rock interactions
- The Rb-normalized SoA and SoG demonstrate that sites downstream of large cities were significantly contaminated
- The Rb-normalized SoA and SoG plots further demonstrate that discharge contribution from Ramganga River has limited impact on the river chemistry of River Ganga
- Though few contamination hotspots were identified, we show that the average composition of the Ramganga River is comparable to the worldwide average river water composition and lower than previous findings in the basin due to the COVID19-related cessation of anthropogenic activities

Further, we highlight the importance of discharge and non-traditional isotopes such as, e.g., Li, Mg, Ca, Ti, V, Cr, Fe, Ni, Cu, Zn, Sr, Ag, Cd, Sn, Pt, and Hg to better understand river chemistry in space and time. This study, therefore, calls for additional assessments of non-traditional stable isotopes in river water samples from India—a way forward to better understand the dynamics of dissolved inorganic constituents in riverine systems.

### Supplementary Information

Below is the link to the electronic supplementary material.

Supplementary file1 (DOCX 1087 KB)

## Data Availability

Full data and supporting information are available as supplementary material.
