# Supplementary material for: Geochemical evolution of dissolved trace elements in space and time in the Ramganga River, India
Source: Environ Monit Assess. 2023 Sep 5;195(10):1150. doi: 10.1007/s10661-023-11665-0 (PMC10480240; doi:10.1007/s10661-023-11665-0)
Supplement: Supplementary file 1 — Supplementary file1 (DOCX 1087 KB) [file 10661_2023_11665_MOESM1_ESM.docx]

**Supplementary information**

**Geochemical evolution of dissolved trace elements along the Ramganga River, India**

**Indra Sekhar Sen*^1^*, Sarwar Nizam^1^, Aqib Ansari*^1^*, Michael Bowes*^2^*, Bharat Choudhary*^1^*, Miriam Glendell*^3^*, Surajit Ray*^4^,* Marian Scott*^4^*, Claire Miller*^4^*, Craig Wilkie*^4^* and Rajiv Sinha*^1^***

***^1^****Department of Earth Sciences, Indian Institute of Technology Kanpur, India*

*^2^ UK Centre for Ecology and Hydrology, Wallingford, OX10 8BB UK*

*^3^The James Hutton Institute, Aberdeen AB15 8QH UK*

*^4^School of Mathematics and Statistics, University of Glasgow, Glasgow G128QQ, UK*

**This file contains**

Figure S1-S2 and their captions.

Table S1–S4 and their captions

**
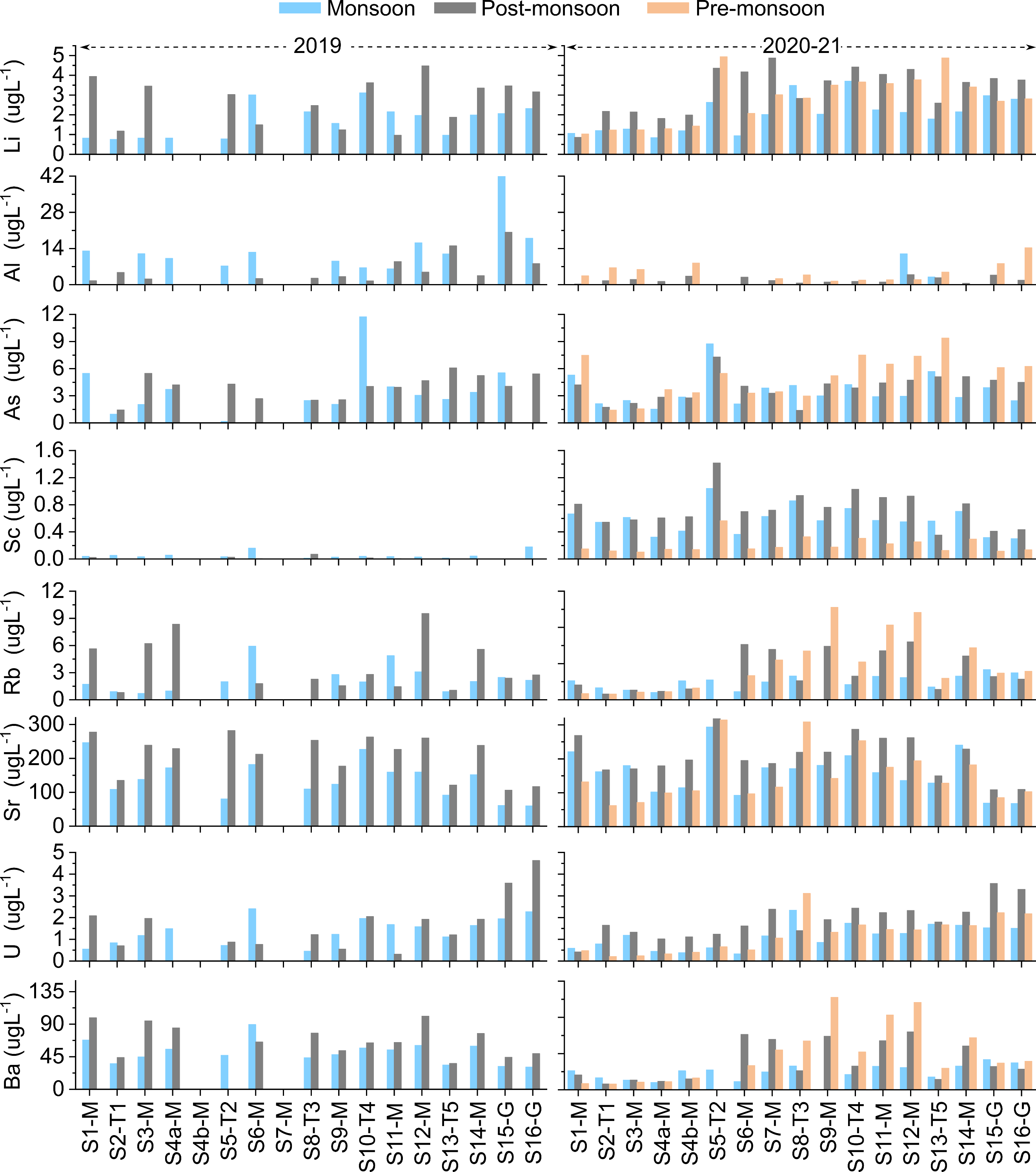
**

**Figure S1.** Dissolved alkali, alkaline metals, Sc, Al, As and U concentration in the RG-T, RG-MS and Ganga River measured during the year 2019 and 2021.


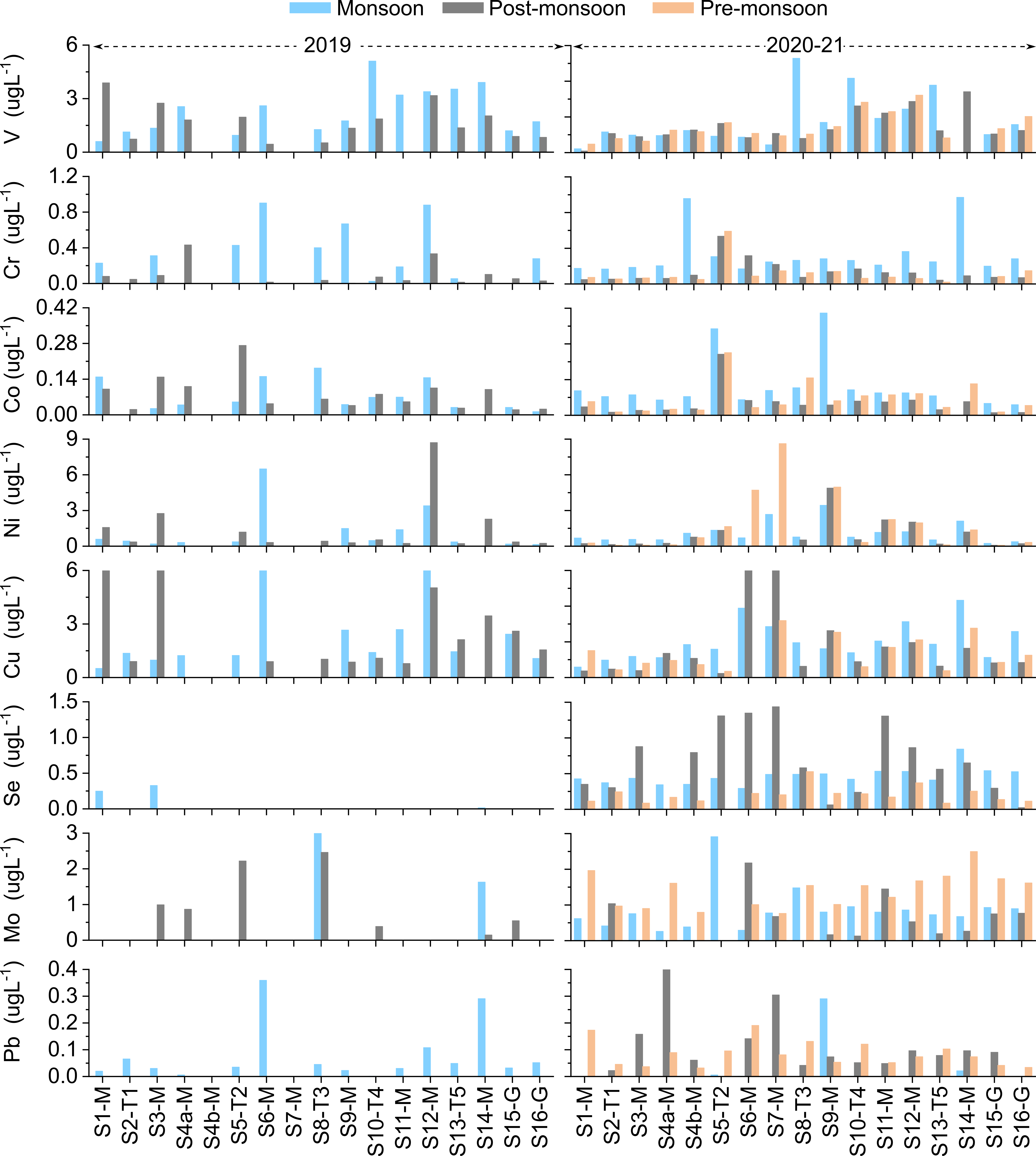


**Figure S2.** Dissolved heavy metal concertation in the RG-T), RG-MS and Ganga River measured during the year 2019 and 2021.

**Table S1.** Detailed information about the sampling sites selected in monitoring the river water quality during the study. Coordinates are given in decimal degree unit.

| **SamID** | **River** | **Location name** | **Latitude(^O^N)** | **Longitude (^o^E)** |
| --- | --- | --- | --- | --- |
| S1-M | Ramganga | Laidarpur-near Sherkote road | 29.30958 | 78.6365 |
| S2-T1 | Khoh | Mukerpuri bila ahatmali | 29.26172 | 78.6277 |
| S3-M | Ramganga | Berkhera Tanda Ahatmali | 29.23915 | 78.6566 |
| S4a-M | Ramganga | Chattapul | 28.89361 | 78.7444 |
| S4b-M | Ramganga | Mokshdham | 28.88139 | 78.7531 |
| S5-T2 | Dhela | Shahpur Mustahkam- Garimat nagar | 28.9325 | 78.8106 |
| S6-M | Ramganga | Katghar | 28.82694 | 78.7989 |
| S7-M | Ramganga | Sikanderpur/ Sikandarpurpatti | 28.7475 | 78.8322 |
| S8-T3 | Kosi | Mansunpur-near rampur | 28.78642 | 78.9868 |
| S9-M | Ramganga | Shahabad | 28.55431 | 79.0454 |
| S10-T4 | Kichha | Thiriya Khetal | 28.46603 | 79.2851 |
| S11-M | Ramganga | Bareilly | 28.29447 | 79.371 |
| S12-M | Ramganga | Shankarpur Kham- near Dataganj | 28.02255 | 79.4857 |
| S13-T5 | Behgul | Kashari Village | 27.69391 | 79.6157 |
| S14-M | Ramganga | Shahjahanpur | 27.49333 | 79.6961 |
| S15-G | Ganga | Farrukhabad | 27.39898 | 79.6276 |
| S16-G | Ganga | Kusumkhor | 27.15006 | 79.886 |

**Table S2:** Trace metal concentration in standard reference material 1643f for water and multi-elemental standards namely Calibration Mix 2 from Agilent and Periodic Table Mix 3 for ICP* from Merck.

| **Element** | **SRM 1643f** | | **Multi-elemental standard** | |
| --- | --- | --- | --- | --- |
|  | Measured (n=14, 1 SD) | Certified | Measured (n=11, 1 SD) | Certified |
|  | µg L^-1^ | µg L^-1^ | µg L^-1^ | µg L^-1^ |
| Li | 16.2 ± 0.51 | 16.59 ± 0.35 | 9.56 ± 0.48 | 10.0 |
| Al | 129 ± 4.3 | 133.8 ± 1.2 | 9.24 ± 1.08 | 10.0 |
| Sc* |  | Not available | 10.4 ± 0.50 | 10.0 |
| V | 34.8 ± 1.12 | 36.07 ± 0.28 | 10.2 ± 0.36 | 10.0 |
| Cr | 18.8 ± 0.77 | 18.50 ± 0.10 | 10.7 ± 0.46 | 10.0 |
| Co | 23.1 ± 0.62 | 25.30 ± 0.17 | 9.9 ± 0.72 | 10.0 |
| Ni | 54.7 ± 1.46 | 59.8 ± 1.4 | 9.75 ± 0.50 | 10.0 |
| Cu | 21.2 ± 0.94 | 21.66 ± 0.71 | 10.3 ± 0.52 | 10.0 |
| Zn | 69.7 ± 2.14 | 74.4 ± 1.7 | 9.69 ± 0.49 | 10.0 |
| As | 52.8 ± 1.1 | 57.42 ± 0.38 | 9.9 ± 0.31 | 10.0 |
| Se | 10.3 ± 0.52 | 11.7 ± 0.081 | 10.4 ± 0.43 | 10.0 |
| Rb | 12.2 ± 0.32 | 12.64 ± 0.13 | 10.7 ± 0.83 | 10.0 |
| Sr | 294 ± 8.59 | 314 ± 19 | 10.0 ± 0.74 | 10.0 |
| Mo | 115 ± 2.87 | 115.3 ± 1.7 | 10.3 ± 0.28 | 10.0 |
| Ba | 497 ± 14.4 | 518.2 ± 7.3 | 10.8 ± 0.32 | 10.0 |
| Pb | 16.9 ± 0.47 | 18.488 ± 0.084 | 9.6 ± 0.42 | 10.0 |
| U |  | Not available | 9.5 ± 0.40 | 10.0 |

| Table S3. Dissolved trace elements measured in individual samples in Ramganga mainstream, its tributaries and Ganga River before after the confluence with Ramganga River. Blank value suggest data below detection limit (BDL). | | | | | | | | | | | | | | | | | |
| --- | --- | --- | --- | --- | --- | --- | --- | --- | --- | --- | --- | --- | --- | --- | --- | --- | --- |
| Sampling | **Sample** | **Li** | **Al** | **Sc** | **V** | **Cr** | **Co** | **Ni** | **Cu** | **As** | **Se** | **Rb** | **Sr** | **Mo** | **Ba** | **Pb** | **U** |
| Season/year | **ID** | **ug/L** | **ug/L** | **ug/L** | **ug/L** | **ug/L** | **ug/L** | **ug/L** | **ug/L** | **ug/L** | **ug/L** | **ug/L** | **ug/L** | **ug/L** | **ug/L** | **ug/L** | **ug/L** |
| *Ramganga mainstream* | | | | | | | | | | | | | | | | | |
| 2019Aug-Mon | S1-M | 0.81 | 13.1 | 0.04 | 0.60 | 0.23 | 0.15 | 0.58 | 0.51 | 5.48 | 0.25 | 1.71 | 246 |  | 68.1 | 0.02 | 0.55 |
| 2019Dec-PM | S1-M | 3.95 | 1.53 | 0.02 | 3.88 | 0.08 | 0.10 | 1.57 | 6.70 |  |  | 5.63 | 278 |  | 98.8 |  | 2.08 |
| 2020Sept-Mon | S1-M | 1.06 |  | 0.67 | 0.21 | 0.17 | 0.10 | 0.68 | 0.59 | 5.28 | 0.43 | 2.10 | 221 | 0.62 | 42.7 |  | 0.59 |
| 2020Dec-PM | S1-M | 0.85 | 0.23 | 0.81 | 0.09 | 0.05 | 0.03 | 0.22 | 0.37 | 4.19 | 0.35 | 1.64 | 269 |  | 80.5 |  | 0.42 |
| 2019Aug-Mon | S3-M | 0.82 | 12.0 | 0.03 | 1.35 | 0.31 | 0.02 | 0.19 | 0.98 | 2.03 | 0.33 | 0.70 | 138 |  | 44.8 | 0.03 | 1.17 |
| 2019Dec-PM | S3-M | 3.45 | 2.20 |  | 2.74 | 0.09 | 0.15 | 2.75 | 6.56 | 5.48 |  | 6.20 | 239 | 0.99 | 94.6 |  | 1.96 |
| 2020Sept-Mon | S3-M | 1.28 |  | 0.61 | 0.98 | 0.19 | 0.08 | 0.57 | 1.18 | 2.48 | 0.44 | 1.06 | 180 | 0.76 | 73.3 |  | 1.19 |
| 2020Dec-PM | S3-M | 2.14 | 1.99 | 0.58 | 0.89 | 0.06 | 0.02 | 0.18 | 0.39 | 2.17 | 0.88 | 1.07 | 170 |  | 51.6 | 0.16 | 1.33 |
| 2019Aug-Mon | S4a-M | 0.82 | 10.2 | 0.06 | 2.55 |  | 0.04 | 0.31 | 1.23 | 3.70 |  | 0.96 | 172 |  | 55.3 | 0.01 | 1.49 |
| 2019Dec-PM | S4a-M |  |  |  | 1.81 | 0.43 | 0.11 |  |  | 4.20 |  | 8.34 | 229 | 0.87 | 84.9 |  |  |
| 2020Sept-Mon | S4a-M | 0.84 |  | 0.32 | 0.96 | 0.20 | 0.06 | 0.54 | 1.12 | 1.52 | 0.34 | 0.80 | 102 | 0.26 | 38.4 |  | 0.45 |
| 2020Dec-PM | S4a-M | 1.81 | 1.23 | 0.61 | 1.00 | 0.06 | 0.02 | 0.24 | 1.36 | 2.84 |  | 0.93 | 179 |  | 54.1 | 0.42 | 1.02 |
| 2019Aug-Mon | S4b-M |  |  |  |  |  |  |  |  |  |  |  |  |  |  |  |  |
| 2019Dec-PM | S4b-M |  |  |  |  |  |  |  |  |  |  |  |  |  |  |  |  |
| 2020Sept-Mon | S4b-M | 1.19 |  | 0.41 | 1.24 | 0.96 | 0.07 | 1.09 | 1.85 | 2.85 | 0.35 | 2.09 | 115 | 0.38 | 52.7 |  | 0.38 |
| 2020Dec-PM | S4b-M | 1.98 | 3.31 | 0.62 | 1.26 | 0.10 | 0.03 | 0.77 | 1.07 | 2.76 | 0.80 | 1.21 | 197 |  | 62.9 | 0.06 | 1.11 |
| 2019Aug-Mon | S6-M | 3.01 | 12.6 | 0.16 | 2.60 | 0.90 | 0.15 | 6.50 | 10.6 |  |  | 5.92 | 182 |  | 89.5 | 0.36 | 2.40 |
| 2019Dec-PM | S6-M | 1.49 | 2.37 | 0.00 | 0.44 | 0.02 | 0.04 | 0.31 | 0.90 | 2.67 |  | 1.78 | 212 |  | 65.4 |  | 0.77 |
| 2020Sept-Mon | S6-M | 0.93 |  | 0.36 | 0.87 | 0.17 | 0.06 | 0.70 | 3.89 | 2.10 | 0.29 | 0.90 | 92.1 | 0.29 | 46.0 | 0.00 | 0.33 |
| 2020Dec-PM | S6-M | 4.17 | 2.90 | 0.70 | 0.84 | 0.32 | 0.06 |  | 6.01 | 4.06 | 1.35 | 6.11 | 195 | 2.18 | 73.4 | 0.14 | 1.61 |
| 2019Aug-Mon | S7-M |  |  |  |  |  |  |  |  |  |  |  |  |  |  |  |  |
| 2019Dec-PM | S7-M |  |  |  |  |  |  |  |  |  |  |  |  |  |  |  |  |
| 2020Sept-Mon | S7-M | 2.01 |  | 0.63 | 0.43 | 0.25 | 0.10 | 2.67 | 2.86 | 3.86 | 0.49 | 1.96 | 174 | 0.77 | 77.4 |  | 1.15 |
| 2020Dec-PM | S7-M | 4.88 | 1.55 | 0.72 | 1.07 | 0.22 | 0.05 |  | 8.00 | 3.28 | 1.44 | 5.56 | 186 | 0.68 | 72.9 | 0.30 | 2.38 |
| 2019Aug-Mon | S9-M | 1.56 | 9.11 | 0.03 | 1.75 | 0.67 | 0.04 | 1.49 | 2.66 | 2.05 |  | 2.78 | 124 |  | 47.9 | 0.02 | 1.23 |
| 2019Dec-PM | S9-M | 1.24 | 3.08 |  | 1.34 | 0.00 | 0.04 | 0.29 | 0.87 | 2.56 |  | 1.56 | 177 |  | 53.5 |  | 0.55 |
| 2020Sept-Mon | S9-M | 2.03 |  | 0.56 | 1.69 | 0.28 | 0.40 | 3.43 | 1.61 | 2.99 | 0.50 |  | 181 | 0.80 | 76.2 | 0.29 | 0.86 |
| Table S3 continued | | | | | | | | | | | | | | | | | |
| Sampling | **Sample** | **Li** | **Al** | **Sc** | **V** | **Cr** | **Co** | **Ni** | **Cu** | **As** | **Se** | **Rb** | **Sr** | **Mo** | **Ba** | **Pb** | **U** |
| Season/year | **ID** | **ug/L** | **ug/L** | **ug/L** | **ug/L** | **ug/L** | **ug/L** | **ug/L** | **ug/L** | **ug/L** | **ug/L** | **ug/L** | **ug/L** | **ug/L** | **ug/L** | **ug/L** | **ug/L** |
| 2020Dec-PM | S9-M | 3.73 | 1.01 | 0.76 | 1.29 | 0.14 | 0.04 | 4.89 | 2.63 | 4.31 | 0.06 | 5.91 | 220 | 0.17 | 67.7 | 0.07 | 1.91 |
| 2019Aug-Mon | S11-M | 2.15 | 6.11 | 0.04 | 3.20 | 0.19 | 0.07 | 1.39 | 2.69 | 3.98 |  | 4.86 | 159 |  | 54.5 | 0.03 | 1.68 |
| 2019Dec-PM | S11-M | 0.97 | 8.93 | 0.01 | 0.01 | 0.03 | 0.05 | 0.23 | 0.78 | 3.93 |  | 1.44 | 226 |  | 64.8 |  | 0.31 |
| 2020Sept-Mon | S11-M | 2.25 |  | 0.57 | 1.92 | 0.21 | 0.09 | 1.16 | 2.04 | 2.90 | 0.53 | 2.60 | 159 | 0.80 | 69.8 |  | 1.25 |
| 2020Dec-PM | S11-M | 4.05 | 1.03 | 0.91 | 2.22 | 0.13 | 0.05 | 2.21 | 1.71 | 4.42 | 1.31 | 5.42 | 261 | 1.45 | 82.8 | 0.05 | 2.23 |
| 2019Aug-Mon | S12-M | 1.97 | 16.2 | 0.03 | 3.39 | 0.88 | 0.15 | 3.39 | 7.09 | 3.05 |  | 3.08 | 160 |  | 60.6 | 0.11 | 1.58 |
| 2019Dec-PM | S12-M | 4.48 | 4.86 |  | 3.17 | 0.33 | 0.11 | 8.70 | 5.03 | 4.67 |  | 9.54 | 260 |  | 101 |  | 1.92 |
| 2020Sept-Mon | S12-M | 2.12 | 12.0 | 0.55 | 2.44 | 0.36 | 0.09 | 1.21 | 3.13 | 2.95 | 0.53 | 2.44 | 136 | 0.86 | 73.2 |  | 1.27 |
| 2020Dec-PM | S12-M | 4.31 | 3.86 | 0.93 | 2.86 | 0.12 | 0.06 | 2.02 | 1.96 | 4.73 | 0.86 | 6.39 | 262 | 0.53 | 88.7 | 0.10 | 2.33 |
| 2019Aug-Mon | S14-M | 1.98 |  | 0.04 | 3.91 |  |  |  |  | 3.39 | 0.01 | 2.02 | 152 | 1.63 | 59.6 | 0.29 | 1.64 |
| 2019Dec-PM | S14-M | 3.36 | 3.50 |  | 2.04 | 0.10 | 0.10 | 2.28 | 3.46 | 5.23 |  | 5.59 | 238 | 0.15 | 77.0 |  | 1.92 |
| 2020Sept-Mon | S14-M | 2.15 |  | 0.70 |  | 0.97 |  | 2.11 | 4.33 | 2.83 | 0.84 | 2.61 | 240 | 0.67 | 65.6 | 0.02 | 1.64 |
| 2020Dec-PM | S14-M | 3.64 | 0.50 | 0.81 | 3.41 | 0.09 | 0.05 | 1.19 | 1.64 | 5.12 | 0.65 | 4.83 | 229 | 0.26 | 78.6 | 0.10 | 2.25 |
| 2021March | S1-M | 1.03 | 3.42 | 0.15 | 0.48 | 0.07 | 0.05 | 0.27 | 1.51 | 7.47 | 0.12 | 0.68 | 132 | 1.97 | 36.9 | 0.17 | 0.47 |
| 2021March | S3-M | 1.24 | 5.89 | 0.10 | 0.64 | 0.07 | 0.02 | 0.08 | 0.80 | 1.56 | 0.09 | 0.84 | 71.1 | 0.90 | 27.6 | 0.04 | 0.24 |
| 2021March | S4a-M | 1.29 |  | 0.14 | 1.26 | 0.07 | 0.02 | 0.12 | 0.96 | 3.68 | 0.17 | 0.89 | 99.5 | 1.61 | 34.4 | 0.09 | 0.33 |
| 2021March | S4b-M | 1.43 | 8.36 | 0.14 | 1.17 | 0.05 | 0.02 | 0.72 | 0.73 | 3.35 | 0.12 | 1.29 | 106 | 0.79 | 34.8 | 0.03 | 0.40 |
| 2021March | S6-M | 2.07 |  | 0.15 | 1.08 | 0.09 | 0.03 | 4.71 |  | 3.30 | 0.22 | 2.68 | 96.7 | 1.01 | 40.6 | 0.19 | 0.51 |
| 2021March | S7-M | 3.02 | 2.33 | 0.17 | 0.94 | 0.15 | 0.04 | 8.62 | 3.19 | 3.45 | 0.20 | 4.39 | 117 | 0.76 | 41.7 | 0.08 | 1.06 |
| 2021March | S9-M | 3.51 | 1.38 | 0.18 | 1.46 | 0.14 | 0.06 | 4.96 | 2.54 | 5.22 | 0.23 | 10.2 | 142 | 1.02 | 49.5 | 0.05 | 1.32 |
| 2021March | S11-M | 3.59 | 1.85 | 0.22 | 2.30 | 0.08 | 0.08 | 2.24 | 1.69 | 6.50 | 0.17 | 8.27 | 175 | 1.22 | 55.6 | 0.05 | 1.45 |
| 2021March | S12-M | 3.77 | 1.97 | 0.25 | 3.22 | 0.06 | 0.09 | 1.96 | 2.11 | 7.38 | 0.37 | 9.66 | 194 | 1.68 | 56.8 | 0.07 | 1.44 |
| 2021March | S14-M | 3.41 |  | 0.29 |  |  | 0.12 | 1.37 | 2.77 |  | 0.25 | 5.75 | 182 | 2.50 | 54.7 | 0.07 | 1.63 |
| *Ramganga Tributaries* | | | | | | | | | | | | | | | | | |
| 2019Aug-Mon | S2-T1 | 0.75 |  | 0.05 | 1.13 |  |  | 0.43 | 1.36 | 0.96 |  | 0.91 | 109 |  | 35.3 | 0.07 | 0.84 |
| 2019Dec-PM | S2-T1 | 1.17 | 4.73 |  | 0.73 | 0.05 | 0.02 | 0.35 | 0.90 | 1.44 |  | 0.80 | 135 |  | 43.9 |  | 0.70 |
| 2020Sept-Mon | S2-T1 | 1.20 |  | 0.54 | 1.16 | 0.17 | 0.07 | 0.53 | 0.98 | 2.11 | 0.37 | 1.31 | 162 | 0.41 | 68.4 |  | 0.79 |
| 2020Dec-PM | S2-T1 | 2.17 | 1.51 | 0.54 | 1.07 | 0.05 | 0.01 | 0.14 | 0.47 | 1.74 | 0.30 | 0.64 | 167 | 1.04 | 49.1 | 0.02 | 1.65 |
| Table S3 continued | | | | | | | | | | | | | | | | | |
| Sampling | **Sample** | **Li** | **Al** | **Sc** | **V** | **Cr** | **Co** | **Ni** | **Cu** | **As** | **Se** | **Rb** | **Sr** | **Mo** | **Ba** | **Pb** | **U** |
| Season/year | **ID** | **ug/L** | **ug/L** | **ug/L** | **ug/L** | **ug/L** | **ug/L** | **ug/L** | **ug/L** | **ug/L** | **ug/L** | **ug/L** | **ug/L** | **ug/L** | **ug/L** | **ug/L** | **ug/L** |
| 2019Aug-Mon | S5-T2 | 0.77 | 7.23 | 0.03 | 0.95 | 0.43 | 0.05 | 0.37 | 1.23 | 0.17 |  | 2.00 | 80.2 |  | 47.0 | 0.04 | 0.72 |
| 2019Dec-PM | S5-T2 | 3.02 |  | 0.03 | 1.96 |  | 0.27 | 1.19 |  | 4.29 |  |  | 282 | 2.22 |  |  | 0.87 |
| 2020Sept-Mon | S5-T2 | 2.63 |  | 1.04 | 0.92 | 0.31 | 0.34 | 1.34 | 1.59 | 8.74 | 0.43 | 2.19 | 294 | 2.91 |  | 0.01 | 0.61 |
| 2020Dec-PM | S5-T2 | 4.36 |  | 1.41 | 1.63 | 0.53 | 0.24 | 1.33 | 0.23 | 7.29 | 1.31 |  | 318 |  |  |  | 1.23 |
| 2019Aug-Mon | S8-T3 | 2.15 |  | 0.01 | 1.26 | 0.40 | 0.18 |  |  | 2.47 |  |  | 109 | 3.22 | 43.6 | 0.04 | 0.45 |
| 2019Dec-PM | S8-T3 | 2.47 | 2.46 | 0.07 | 0.52 | 0.04 | 0.06 | 0.42 | 1.03 | 2.52 |  | 2.28 | 253 | 2.46 | 77.7 |  | 1.22 |
| 2020Sept-Mon | S8-T3 | 3.49 |  | 0.86 | 5.28 | 0.27 | 0.11 | 0.77 | 1.95 | 4.14 | 0.49 | 2.61 | 171 | 1.48 | 43.6 |  | 2.34 |
| 2020Dec-PM | S8-T3 | 2.83 | 0.63 | 0.94 | 0.80 | 0.07 | 0.04 | 0.52 | 0.63 | 1.38 | 0.58 | 2.09 | 219 |  | 56.4 | 0.04 | 1.40 |
| 2019Aug-Mon | S10-T4 | 3.11 | 6.56 | 0.04 | 5.10 | 0.03 | 0.07 | 0.48 | 1.41 | 11.7 |  | 1.98 | 226 |  | 56.9 | 0.00 | 1.96 |
| 2019Dec-PM | S10-T4 | 3.63 | 1.47 | 0.02 | 1.86 | 0.07 | 0.08 | 0.54 | 1.08 | 4.04 |  | 2.80 | 263 | 0.38 | 64.3 |  | 2.05 |
| 2020Sept-Mon | S10-T4 | 3.70 |  | 0.75 | 4.17 | 0.26 | 0.10 | 0.76 | 1.39 | 4.23 | 0.42 | 1.68 | 209 | 0.95 | 67.6 | 0.00 | 1.74 |
| 2020Dec-PM | S10-T4 | 4.43 | 1.22 | 1.03 | 2.62 | 0.17 | 0.06 | 0.55 | 0.89 | 3.87 | 0.24 | 2.61 | 287 | 0.13 | 60.5 | 0.05 | 2.43 |
| 2019Aug-Mon | S13-T5 | 0.96 | 11.9 | 0.01 | 3.53 | 0.05 | 0.03 | 0.36 | 1.45 | 2.60 |  | 0.90 | 91.7 |  | 33.5 | 0.05 | 1.11 |
| 2019Dec-PM | S13-T5 | 1.87 | 15.0 |  | 1.37 | 0.01 | 0.03 | 0.22 | 2.12 | 6.08 |  | 1.05 | 121 |  | 35.7 |  | 1.21 |
| 2020Sept-Mon | S13-T5 | 1.78 | 3.03 | 0.56 | 3.78 | 0.25 | 0.08 | 0.53 | 1.87 | 5.69 | 0.41 | 1.42 | 129 | 0.73 | 62.0 |  | 1.70 |
| 2020Dec-PM | S13-T5 | 2.59 | 2.65 | 0.35 | 1.22 | 0.04 | 0.02 | 0.18 | 0.64 | 5.10 | 0.56 | 1.14 | 150 | 0.20 | 30.2 | 0.08 | 1.79 |
| 2020March | S2-T1 | 1.23 | 6.53 | 0.12 | 0.79 | 0.06 | 0.01 | 0.08 | 0.44 | 1.41 | 0.24 | 0.63 | 62.1 | 0.97 | 25.4 | 0.05 | 0.21 |
| 2020March | S5-T2 | 4.95 |  | 0.56 | 1.68 | 0.59 | 0.25 | 1.65 | 0.35 | 5.48 |  |  | 315 |  | 133.5 | 0.10 | 0.65 |
| 2020March | S8-T3 | 2.85 | 3.77 | 0.33 | 1.04 | 0.13 | 0.15 |  |  | 2.97 | 0.53 | 5.39 | 309 | 1.55 | 74.3 | 0.13 | 3.11 |
| 2020March | S10-T4 | 3.66 | 1.73 | 0.31 | 2.82 | 0.06 | 0.08 | 0.32 | 0.61 | 7.51 | 0.22 | 4.17 | 253 | 1.54 | 45.8 | 0.12 | 1.66 |
| 2020March | S13-T5 | 4.88 | 4.87 | 0.13 | 0.83 | 0.02 | 0.03 | 0.11 | 0.39 | 9.38 | 0.09 | 2.38 | 128 | 1.81 | 25.2 | 0.10 | 1.66 |
| *Ganga River* | | | | | | | | | | | | | | | | | |
| 2019Aug-Mon | S15-G | 2.06 | 41.9 |  | 1.20 |  | 0.03 |  | 2.43 | 5.53 |  | 2.47 | 60.9 |  | 31.7 | 0.03 | 1.94 |
| 2019Dec-PM | S15-G | 3.47 | 20.3 |  | 0.88 | 0.05 | 0.02 | 0.18 | 2.60 | 4.04 |  | 2.39 | 106 | 0.54 | 44.3 |  | 3.59 |
| 2020Sept-Mon | S15-G | 2.97 |  | 0.32 | 1.01 | 0.20 | 0.05 | 0.36 | 1.13 | 3.89 | 0.54 | 3.34 | 69.2 | 0.93 | 41.6 |  | 1.53 |
| 2020Dec-PM | S15-G | 3.84 | 3.69 | 0.41 | 1.04 | 0.07 | 0.01 | 0.24 | 0.82 | 4.71 | 0.30 | 2.57 | 109 | 0.75 | 41.7 | 0.09 | 3.57 |
| 2019Aug-Mon | S16-G | 2.32 | 18.0 | 0.18 | 1.71 | 0.28 | 0.01 | 0.14 | 1.07 |  |  | 2.15 | 59.8 |  | 30.7 | 0.05 | 2.27 |
| 2019Dec-PM | S16-G | 3.16 | 8.12 |  | 0.83 | 0.03 | 0.02 | 0.24 | 1.55 | 5.42 |  | 2.74 | 117 |  | 49.3 |  | 4.63 |
| 2020Sept-Mon | S16-G | 2.79 |  | 0.30 | 1.57 | 0.28 | 0.04 | 0.37 | 2.58 | 2.45 | 0.53 | 2.98 | 68.3 | 0.90 | 39.6 |  | 1.51 |
| Table S3 continued | | | | | | | | | | | | | | | | | |
| Sampling | **Sample** | **Li** | **Al** | **Sc** | **V** | **Cr** | **Co** | **Ni** | **Cu** | **As** | **Se** | **Rb** | **Sr** | **Mo** | **Ba** | **Pb** | **U** |
| Season/year | **ID** | **ug/L** | **ug/L** | **ug/L** | **ug/L** | **ug/L** | **ug/L** | **ug/L** | **ug/L** | **ug/L** | **ug/L** | **ug/L** | **ug/L** | **ug/L** | **ug/L** | **ug/L** | **ug/L** |
| 2020Dec-PM | S16-G | 3.76 | 1.69 | 0.43 | 1.24 | 0.07 | 0.01 | 0.21 | 0.84 | 4.48 | 0.02 | 2.27 | 110 | 0.77 | 39.2 |  | 3.30 |
| 2021March | S15-G | 2.69 | 8.18 | 0.12 | 1.34 | 0.08 | 0.01 | 0.08 | 0.85 | 6.13 | 0.14 | 2.95 | 85.6 | 1.74 | 27.9 | 0.04 | 2.22 |
| 2021March | S16-G | 2.81 | 14.3 | 0.14 | 2.02 | 0.15 | 0.04 | 0.32 | 1.25 | 6.25 | 0.11 | 3.15 | 103 | 1.62 | 30.2 | 0.03 | 2.18 |

**Table S4.** Comparison of average dissolved trace element concentrations in major Indian and global rivers with the Ramganga River.

| Study area | Li | Al | Sc | V | Cr | Co | Ni | Cu | As | Se | Rb | Sr | Mo | Ba | Pb | U | Reference |
| --- | --- | --- | --- | --- | --- | --- | --- | --- | --- | --- | --- | --- | --- | --- | --- | --- | --- |
| Ramganga mainstream | 2.31 | 5.01 | 0.37 | 1.66 | 0.23 | 0.08 | 1.93 | 2.86 | 3.77 | 0.47 | 3.61 | 180 | 0.95 | 62.6 | 0.11 | 1.24 | This study |
| RG-T | 2.67 | 4.71 | 0.42 | 1.93 | 0.18 | 0.10 | 0.57 | 1.05 | 4.29 | 0.44 | 1.95 | 194 | 1.37 | 53.6 | 0.06 | 1.36 |  |
| Ganga before confluence | 3.00 | 18.5 | 0.28 | 1.10 | 0.10 | 0.02 | 0.21 | 1.57 | 4.86 | 0.32 | 2.74 | 86.1 | 0.99 | 37.4 | 0.05 | 2.57 |  |
| Ganga after confluence | 2.97 | 10.5 | 0.26 | 1.47 | 0.16 | 0.03 | 0.26 | 1.46 | 4.65 | 0.22 | 2.66 | 91.6 | 1.09 | 37.8 | 0.04 | 2.78 |  |
| Ramganga mainstream | 4.37 | 15.2 |  | 7.86 | 1.00 | 0.43 | 4.83 | 6.27 | 18.2 |  | 8.70 | 231 |  | 103 | 0.48 |  | (Boral et al., 2020) |
| Ramganga mainstream |  |  |  |  |  |  | 14.9 | 39 |  |  |  | 183 |  |  | 13.1 |  | (Gurjar and Tare, 2019) |
| Ramganga tributary |  |  |  |  |  |  | 19.8 | 59 |  |  |  | 239 |  |  | 54.4 |  |  |
| Ramganga mainstream) |  |  |  |  |  |  |  |  | 0.13 |  |  |  |  |  | 0.89 |  | (Sarah et al., 2019) |
| Ramganga mainstream |  |  |  |  |  |  |  |  |  |  |  |  |  |  | 5.1 |  | (Khan et al., 2017) |
| Ganga downstream | 5.67 | 17.57 |  | 7.47 | 0.92 | 0.22 | 2.24 | 2.88 | 8.66 |  | 7.57 | 203 |  | 67..2 | 0.48 |  | (Boral et al., 2020) |
| Ganga upstream | 4.7 | 25.6 |  | 0.4 | 0.8 | 0.2 | 1.70 | 2.7 | 5.80 |  | 5 | 58.5 |  | 19.2 | 0.4 |  |  |
| Yamuna River, India | 4.37 | 15.23 |  | 7.87 | 1.0 | 0.43 | 4.83 | 6.27 | 18.2 |  | 8.70 | 231 |  | 103 | 0.37 |  |  |
| Gomti River, India | 10.2 | 10.7 |  | 10.8 | 1 | 0.43 | 2.87 | 3.93 | 17.3 |  | 6.63 | 416 |  | 110 | 0.5 |  |  |
| Kosi River, India | 3.77 | 36.7 |  | 2.7 | 0.83 | 0.13 | 1.7 | 2.27 | 4.70 |  | 5.43 | 90.8 |  | 30.3 | 0.27 |  |  |
| Gandaki, central Nepal | 60.6 |  | 2.04 | 0.48 | 3.02 | 0.55 | 1.41 | 1.89 | 2.02 |  | 20.1 | 322 |  | 38.7 | 0.61 | 4.57 | (Pant et al., 2020) |
| Indus River, China | 135 | <8.8 |  |  | 2 |  |  | 0.40 | 13.7 |  | 1.9 | 89.8 |  | 5.6 |  | 1.6 | (Qu et al., 2019) |
| Yarlung Tsangpo, China | 33 | 20.6 |  |  | 2.7 |  |  | 1.40 | 10.5 |  | 3.4 | 150 | 1.2 | 12 | 5.6 | 2.2 |  |
| Yangtze River, China |  |  |  | 10.5 | 20.9 |  | 13.4 | 10.7 | 13.2 | 114 |  | 210 |  | 37.4 | 55.1 |  | (Wu et al., 2009) |
| Tarim River, China | 65.4 | 84.3 |  | 1.06 | 0.43 | 0.1 | 1.79 | 1.22 | 3.07 |  |  | 1731 |  | 53.0 | 0.45 |  | (Xiao et al., 2014) |
| Amazon River, Mexico |  |  |  | 0.85 | 0.55 | 0.04 | 0.61 | 1.04 |  |  | 1.89 | 35.8 | 0.19 | 22.5 |  | 0.04 | (Seyler and Boaventura, 2003) |
| Thames River, UK | 2 | 46 |  |  |  |  |  |  |  |  |  | 117 |  | 95 |  |  | (Neal et al., 2012) |
| Stream River, Ghana |  | 18 |  |  | 2.65 | 0.52 | 0.85 |  | 30.0 |  |  | 0.46 |  | 0.04 | 1.4 |  | (Asante et al., 2007) |
| Sava River, Croatia |  |  |  |  | 0.32 | 0.06 | 0.59 | 0.54 |  |  |  |  |  |  | 0.06 |  | (Dragun et al., 2009) |
| Seine River, France |  |  |  |  |  |  |  | 2.23 | 0.75 |  | 1.53 | 690 | 0.68 | 27 | 0.21 |  | (Elbaz-Poulichet et al., 2006) |
| Trinity River USA |  | 4.81 |  | 2.05 |  | 0.16 | 2.07 | 1.15 |  |  |  |  |  |  | 0.03 |  | (Warnken and Santschi, 2009) |
| Mississipi (downstream) | 8 | 4531 |  | 11 |  |  |  |  | 12.0 |  |  | 165 |  | 97 |  |  | (Reiman et al., 2018) |
| Meghna River, Bangladesh |  |  |  |  | 14.5 |  | 5 |  | 11.3 |  |  |  |  |  | 4.4 |  | (Islam et al., 2020) |
| Rhône River, Switzerland | 3.89 |  |  | 0.7 |  |  | 0.98 | 2.08 | 1.9 |  | 1.3 | 360 | 0.84 | 31.9 | 0.06 | 0.88 | (Olivier et al. 2011) |
| Guadalquivir River, Spain |  |  |  |  |  | 0.15 | 2.31 | 2.64 |  |  |  |  |  |  | 0.18 |  | (Mendiguchía et al., 2007) |
| Global river average | 1.84 | 32 | 1.2 | 0.71 | 0.7 | 0.15 | 0.8 | 1.48 | 0.62 |  | 1.63 | 60 | 0.42 | 23 | 0.08 | 0.37 | (Viers et al., 2009) |

**References**

Asante, K.A., Agusa, T., Subramanian, A., Ansa-Asare, O.D., Biney, C.A., Tanabe, S., 2007. Contamination status of arsenic and other trace elements in drinking water and residents from Tarkwa, a historic mining township in Ghana. Chemosphere 66, 1513–1522. https://doi.org/10.1016/j.chemosphere.2006.08.022

Boral, S., Sen, I.S., Tripathi, A., Sharma, B., Dhar, S., 2020. Tracking Dissolved Trace and Heavy Metals in the Ganga River From Source to Sink: A Baseline to Judge Future Changes. Geochemistry, Geophys. Geosystems 21, 1–22. https://doi.org/10.1029/2020GC009203

Dragun, Z., Roje, V., Mikac, N., Raspor, B., 2009. Preliminary assessment of total dissolved trace metal concentrations in Sava River water. Environ. Monit. Assess. 159, 99–110. https://doi.org/10.1007/s10661-008-0615-9

Elbaz-Poulichet, F., Seidel, J.L., Casiot, C., Tusseau-Vuillemin, M.H., 2006. Short-term variability of dissolved trace element concentrations in the Marne and Seine Rivers near Paris. Sci. Total Environ. 367, 278–287. https://doi.org/10.1016/j.scitotenv.2005.11.009

Gurjar, S.K., Tare, V., 2019. Spatial-temporal assessment of water quality and assimilative capacity of river Ramganga, a tributary of Ganga using multivariate analysis and QUEL2K. J. Clean. Prod. 222, 550–564. https://doi.org/10.1016/j.jclepro.2019.03.064

Islam, A.R.M.T., Islam, H.M.T., Mia, M.U., Khan, R., Habib, M.A., Bodrud-Doza, M., Siddique, M.A.B., Chu, R., 2020. Co-distribution, possible origins, status and potential health risk of trace elements in surface water sources from six major river basins, Bangladesh. Chemosphere 249. https://doi.org/10.1016/j.chemosphere.2020.126180

Khan, M.Y.A., Gani, K.M., Chakrapani, G.J., 2017. Spatial and temporal variations of physicochemical and heavy metal pollution in Ramganga River—a tributary of River Ganges, India. Environ. Earth Sci. 76, 1–13. https://doi.org/10.1007/s12665-017-6547-3

Mendiguchía, C., Moreno, C., García-Vargas, M., 2007. Evaluation of natural and anthropogenic influences on the Guadalquivir River (Spain) by dissolved heavy metals and nutrients. Chemosphere 69, 1509–1517. https://doi.org/10.1016/j.chemosphere.2007.05.082

Neal, C., Bowes, M., Jarvie, H.P., Scholefield, P., Leeks, G., Neal, M., Rowland, P., Wickham, H., Harman, S., Armstrong, L., Sleep, D., Lawlor, A., Davies, C.E., 2012. Lowland river water quality: a new UK data resource for process and environmental management analysis. Hydrol. Process. 26, 949–960. https://doi.org/10.1002/hyp.8344

Pant, R.R., Zhang, F., Rehman, F.U., Koirala, M., Rijal, K., Maskey, R., 2020. Spatiotemporal characterization of dissolved trace elements in the Gandaki River, Central Himalaya Nepal. J. Hazard. Mater. 389, 121913. https://doi.org/10.1016/j.jhazmat.2019.121913

Qu, B., Zhang, Y., Kang, S., Sillanpää, M., 2019. Water quality in the Tibetan Plateau: Major ions and trace elements in rivers of the “Water Tower of Asia.” Sci. Total Environ. 649, 571–581. https://doi.org/10.1016/j.scitotenv.2018.08.316

Reiman, J.H., Xu, Y.J., He, S., DelDuco, E.M., 2018. Metals geochemistry and mass export from the Mississippi-Atchafalaya River system to the Northern Gulf of Mexico. Chemosphere 205, 559–569. https://doi.org/10.1016/j.chemosphere.2018.04.094

Sarah, R., Tabassum, B., Idrees, N., Hashem, A., Abd_Allah, E.F., 2019. Bioaccumulation of heavy metals in Channa punctatus (Bloch) in river Ramganga (U.P.), India. Saudi J. Biol. Sci. 26, 979–984. https://doi.org/10.1016/j.sjbs.2019.02.009

Seyler, P.T., Boaventura, G.R., 2003. Distribution and partition of trace metals in the Amazon basin. Hydrol. Process. 17, 1345–1361. https://doi.org/10.1002/hyp.1288

Viers, J., Dupré, B., Gaillardet, J., 2009. Chemical composition of suspended sediments in World Rivers: New insights from a new database. Sci. Total Environ. 407, 853–868. https://doi.org/10.1016/j.scitotenv.2008.09.053

Warnken, K.W., Santschi, P.H., 2009. Delivery of trace metals (Al, Fe, Mn, V, Co, Ni, Cu, Cd, Ag, Pb) from the Trinity River watershed towards the ocean. Estuaries and Coasts 32, 158–172. https://doi.org/10.1007/s12237-008-9088-x

Wu, B., Zhao, D.Y., Jia, H.Y., Zhang, Y., Zhang, X.X., Cheng, S.P., 2009. Preliminary risk assessment of trace metal pollution in surface water from Yangtze River in Nanjing section, China. Bull. Environ. Contam. Toxicol. 82, 405–409. https://doi.org/10.1007/s00128-008-9497-3

Xiao, J., Jin, Z., Wang, J., 2014. Geochemistry of trace elements and water quality assessment of natural water within the Tarim River Basin in the extreme arid region, NW China. J. Geochemical Explor. 136, 118–126. https://doi.org/10.1016/j.gexplo.2013.10.013
